# Acidosis Forces Fatty Acid Uptake and Metabolism in Cancer Cells Regardless of Genotype

**DOI:** 10.1002/advs.202505436

**Published:** 2025-07-29

**Authors:** Sébastien Ibanez, Maria Virginia Giolito, Sultan Al‐Siyabi, Kristian Serafimov, Florine Laloux, Emeline Dierge, Léo Aubert, Céline Guilbaud, Selena Kaye, Paula Varon, Dorothée Marchand, Hanne Vlieghe, Emmanuel Hermans, Caroline Bouzin, Davide Brusa, Christiani Andrade Amorim, Barabara Pachikian, Yvan Larondelle, Cyril Corbet, Chantal Dessy, Olivier Feron

**Affiliations:** ^1^ Pole of Pharmacology and Therapeutics (FATH) Institut de Recherche Expérimentale et Clinique (IREC), UCLouvain Brussels B‐1200 Belgium; ^2^ IREC Metabolomics Core, UCLouvain Brussels B‐1200 Belgium; ^3^ Louvain Institute of Biomolecular Science and Technology (LIBST), UCLouvain Louvain‐la‐Neuve B‐1348 Belgium; ^4^ Pôle de Recherche en Physiopathologie de la Reproduction Institut de Recherche Expérimentale et Clinique (IREC) UCLouvain Brussels B‐1200 Belgium; ^5^ Group of Neuropharmacology Institute of Neuroscience, UCLouvain Brussels B‐1200 Belgium; ^6^ Imaging Platform 2IP Institut de Recherche Expérimentale et Clinique (IREC), UCLouvain Brussels B‐1200 Belgium; ^7^ CytoFlux‐Flow Cytometry and Cell Sorting Platform Institut de Recherche Expérimentale et Clinique (IREC), UCLouvain Brussels B‐1200 Belgium; ^8^ Center of Investigation in Clinical Nutrition, UCLouvain Louvain‐la‐Neuve B‐1348 Belgium; ^9^ Walloon Excellence in Life Sciences and BIOtechnology (WELBIO) department WEL Research Institute Wavre B‐1300 Belgium

**Keywords:** acidosis, cancer, fatty acid, lipid metabolism, peroxisome

## Abstract

While proteins facilitate fatty acid (FA) partitioning into plasma membranes, movement between membrane leaflets occurs through a “flip‐flop” mechanism. This study provides evidence that biological acidosis, as encountered in tumors and ischemic diseases, promotes FA protonation, thereby enhancing neutral, non‐ionized FA uptake. This positions the altered lipid metabolism in acid‐exposed cells as a consequence, rather than a cause, of preferential FA uptake. Cancer cell vulnerability, independent of their genetic background, directly stems from this paradigm shift, as detoxifying the overload of very long‐chain FA (VLCFA) becomes highly dependent on peroxisomal activity. Inhibition of peroxisomal function in acid‐exposed cancer cells leads to the rerouting of these fatty acids into triglycerides within lipid droplets, but also into phospholipids, contributing to membrane alterations, triggering ER stress, and ultimately supporting cytotoxicity. Using patient‐derived tumor organoids and sera from human volunteers supplemented with polyunsaturated FA (PUFA), it is shown that inhibiting peroxisomal ACOX1 selectively kills acid‐exposed cancer cells, an effect exacerbated by pharmacological stimulation of glycolysis. Similar acid‐driven FA uptake is observed in endothelial cells and cardiac myocytes, opening new therapeutic avenues not only cancer but also cardiovascular diseases.

## Graphical abstract

This study demonstrates that biological acidosis, commonly observed in tumors and ischemic conditions, actively promotes the protonation of fatty acids, thereby facilitating the uptake of their neutral forms. Consequently, the preference for lipid metabolism in acid‐exposed cells does not arise from genetic reprogramming that prioritizes lipids as the primary energy source, but rather from the direct effect of enhanced fatty acid uptake.

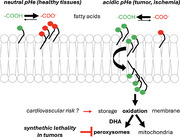

## 1 Introduction

A vivid debate surrounding the involvement of proteins in fatty acid (FA) transport across the plasma membrane has animated the scientific community for decades.^[^ ^1^, ^2^, ^3^, ^4^, ^5^ ^]^ A reasonable consensus emerged lately claiming that while proteins may contribute to the cellular uptake of FAs or their metabolism, once FAs are in a membrane, their transmembrane movement may occur by passive diffusion. Proteins such as CD36 and FABP function as facilitators, accelerating FA partitioning into the lipid bilayer and promoting desorption on the inner side of the membrane, respectively.^[^ ^6^, ^7^, ^8^, ^9^, ^10^ ^]^ In this model, a less contentious flip‐flop mechanism describes the translocation of FAs from the outer to the inner leaflet.^[^ ^11^, ^12^, ^13^ ^]^ While the repositioning of the intercalated FA hydrocarbon chain between phospholipids from the outer to the inner leaflet of the lipid bilayer is plausible, the transfer of the carboxyl head group from the aqueous interface of the interstitial fluid to the opposite cytosolic interface is less straightforward. It is proposed that the neutral, non‐ionized form of FAs traverses the membrane by swiftly transitioning to the inner leaflet.^[^ ^14^ ^]^ The desorption is then associated with the re‐establishment of ionization equilibrium, resulting in the release of H^+^ into the internal compartment.^[^ ^15^ ^]^

The apparent pKa values in the hydrophobic environment of plasma membranes are usually reported to be above 7 so that approximately half of FA are protonated at physiological extracellular pH (pHe).^[^ ^1^, ^11^, ^14^, ^16^ ^]^ Although biophysical studies have shown that changes in pHe can alter the proportion of protonated FAs,^[^ ^11^ ^]^ the role of reduced pHe in pathological conditions as a driver of preferential FA uptake and downstream metabolism has not been addressed. Local acidosis has however been associated with ischemic cardiovascular tissues and tumor tissues for over a century.^[^ ^17^, ^18^ ^]^ While ischemia‐driven hypoxia (and reoxygenation effects upon revascularization) have largely overshadowed the impact of acidosis in cardiovascular diseases, tumor acidosis has gained prominence in recent decades through its recognition as a cancer hallmark,^[^ ^19^, ^20^ ^]^ significantly independent of hypoxia.^[^ ^21^ ^]^ A link between the acidic regions in tumors, with a mean pHe around 6.5, and an increase in the proportion of non‐ionized FAs, which would critically enhance their transport across the plasma membrane, is yet more relevant that we and others documented that acid‐exposed cancer cells exhibit a dramatic shift from glucose to FA metabolism,^[^ ^22^, ^23^, ^24^ ^]^ supporting an invasive and pro‐metastatic phenotype.^[^ ^19^, ^20^, ^25^ ^]^ A highly favorable FA gradient in acid‐exposed cancer cells has thus the potential to be supported by the increased FA protonation and the rapid diversion of membrane‐impermeable fatty acylCoA from returning to the plasma membrane. Such biophysical driving force for enhanced FA diffusion could represent a vulnerability for cancer cells facing tumor acidosis. One may indeed hypothesize that some downstream intracellular mechanisms of FA handling may reach saturation and sensitize cancer cells to treatments that promote lipotoxicity. This extends beyond our previous work, where we demonstrated that PUFA supplementation can exploit the altered lipid metabolism of acid‐exposed cancer cells to induce ferroptosis.^[^ ^26^ ^]^ Indeed, we showed that inhibition of lipid droplet (LD) formation (using diacylglycerol acyltransferase (DGAT) inhibitors) was necessary for this strategy to be effective at PUFA concentrations likely achievable in tumors. A more detailed understanding of the mechanisms driving enhanced FA handling under acidosis may therefore reveal new vulnerabilities that could be therapeutically targeted, independently of ferroptosis and potentially using other classes of drugs.

Here, we found that FA delivery is dramatically enhanced in cancer cells cultured at an acidic pH as well as in more biologically relevant conditions such in response to hypoxia or exposure to MPC inhibitor,^[^ ^27^ ^]^ both promoting a high glycolytic turnover and reduction in pHe. We also showed that as far as PUFAs are concerned, the high FA delivery in acid‐exposed cancer cells leads to peroxisomal activation. This prompted us to identify the inhibition of peroxysomal ACOX1 activity as a selective mode of acid‐exposed cancer cell killing. Remarkably, this effect was enhanced by docosahexaenoic acid (DHA) supplementation in both mice and human volunteers, in a ferroptosis‐independent manner. Finally, we also provide evidence that the acidosis‐triggered preferential FA diffusion unraveled in cancer cells also applies to endothelial and cardiac cells, thereby broadening the paradigm to ischemic diseases and opening new avenues of investigation.

## 2 Results

### 2.1 Extracellular pH Drop Determines the Extent of FA Uptake Independently of Cancer Cell Type

**Figure 1:**
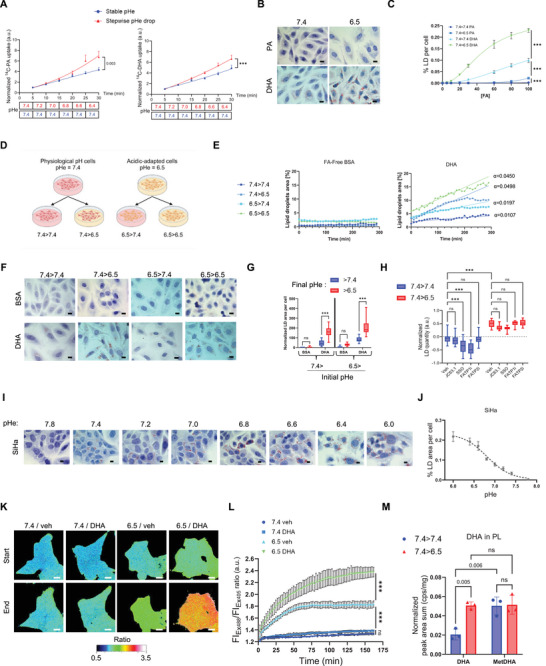
Extracellular pHe determines the extent of fatty acid (FA) uptake. A) Normalised quantification of ^14^C FA uptake during stepwise medium acidification in SiHa cells (vs maintaining stable pHe) for palmitic acid (PA) (left graph) and docosahexaenoic acid (DHA) (right graph) (N = 3, n = 1). B,C) Representative pictures B) and quantification C) of ORO‐stained lipid droplets (LD) in SiHa cells maintained at pHe 7.4 (7.4 > 7.4) or shifted to pHe 6.5 (7.4 > 6.5) upon supplementation for 24 h with the indicated concentration ranges of PA or DHA (N = 3, n = 5 fields). D) Schematic protocol of pHe swap in cancer cells maintained at physiological pHe (7.4) or chronically adapted to acidic pHe (6.5). E) Time‐course quantification of LD accumulation (expressed as a ratio to LD content at time 0) in SiHa performed using Nanolive imaging after supplementation with 25 µm DHA or FA‐free BSA as vehicle (N = 1, n = 2). α = early phase slope values (i.e., before the plateau). F,G) Representative end‐point pictures F) and quantification G) of LD in SiHa cells maintained at the indicated pHe (7.4 > 7.4 and 6.5 > 6.5) or following pHe swapping (7.4 > 6.5 and 6.5 > 7.4) and supplemented with 50 µm DHA or FA‐free BSA as vehicle (N = 2, n = 10 fields). H) Quantification of BODIPY‐stained LD in SiHa cells maintained in 7.4 (7.4 > 7.4) or shifted in 6.5 (7.4 > 6.5) upon supplementation for 6 h with DHA and coupled with FA transporter inhibitors (2 µg mL^−1^ JC63.1, 10 µm SSO, 2 µm FATP1i, 2 µm FATP2i) or DMSO as vehicle (N = 3, n = 5). I,J) Representative pictures I) and quantification J) of ORO‐stained LD in SiHa cells exposed to the indicated pHe and supplemented with 50 µm DHA or FA‐free BSA as vehicle (N = 2, n = 5 fields). K,L) Representative pictures K) and quantification L) of pHi in HCT116 cells transduced with a ratiometric pH‐sensitive protein (pHluorin2) and exposed for 160 min to 50 µM DHA or FA‐free BSA as vehicle (N = 3, n = 1). M. HPLC‐MS quantification of DHA esterified in phospholipids from SiHa cells maintained at 7.4 (7.4 > 7.4) or shifted to 6.5 (7.4 > 6.5) and supplemented with 50 µm DHA or Methyl‐DHA for 24 h (N = 3, n = 1). All scale bars: 10 µm. Vehicles consist of an equivalent concentration of FA‐free BSA for DHA or DMSO for the drugs. Graphs are presented as mean ± SEM (A, C, J, L), min to max whisker plots, range = Q1 to Q3, and line = median G,H) or mean ± SD (M). N = biological replicates, and n = technical replicates. ns = non‐significant; ***p < 0.001. Significance was determined by two‐way ANOVA with Tukey's multiple comparison test. The *p*‐values reported for A and C refer to the column factor, whereas those in L indicate the differences between the final points of the kinetics.

To assess the extracellular pH (pHe) capacity to modulate FA uptake, we used ^14^C‐labelled FAs to perform a short‐time kinetic experiment where the pHe is decreased stepwise over time. The uptake of saturated palmitic acid (PA) and polyunsaturated docosahexaenoic acid (DHA) consistently increased under conditions where the pHe was decreased in both cervix SiHa (**Figure** **1**A) and laryngeal FaDu cancer cells (Figure S1A, Supporting Information). We previously reported that in acidic cancer cells, the rate of palmitate beta‐oxidation was approximately 10‐fold higher than that of DHA which is instead predominantly stored intact within lipid droplets (LD).^[^ ^26^ ^]^ Accordingly, in the subsequent experiments, we focused on DHA, utilizing LD accumulation as a surrogate marker for the dynamic FA uptake induced by acidic pHe, rather than tracking the oxidized products of PA and the multiple metabolic fates of acetyl‐CoA. For this purpose, we used cancer cells either maintained at physiological pHe 7.4 or chronically adapted to acidic pHe 6.5, followed by a subsequent acute pHe swap. Oil red O (ORO) staining revealed that LD accumulated in proportion to the DHA uptake in a larger extent at acidic pHe (than pHe 7.4) whereas an increase in LD formation was only detectable at the highest PA concentrations at pHe 6.5 (Figure 1B,C; Figure S1B,C, Supporting Information). To track live accumulation of LD following DHA exposure in cells undergoing an acutely swapped pHe (Figure 1D), we then used Nanolive system that exploits holotomography to capture label‐free timelapse images of LD dynamics. Interestingly, cells swapped from physiological to acidic pHe (7.4 > 6.5) increased LD formation upon exposure to 50 µM DHA (vs cells maintained at pHe 7.4) while cells swapped the other way around (6.5 > 7.4) significantly reduced the extent of LD formation (vs cells maintained at pHe 6.5) (Figure 1E). Measurement of LD formation slopes confirmed that in the first hour after DHA exposure, velocity of LD formation in 7.4 > 6.5 condition reached the value determined in cells maintained at acidic pHe (6.5 > 6.5) and inversely, velocity measured in 6.5 > 7.4 cells got closer to that of cells maintained at physiological pHe (7.4 > 7.4) (see values in Figure 1E). ORO staining confirmed the enhanced LD formation in 7.4 > 6.5 SiHa cells and the significantly lesser LD accumulation in 6.5 > 7.4 SiHa cells (Figure 1F and quantification in Figure 1G). These results were confirmed in three other cancer cell lines from different origins, namely laryngeal FaDu, colorectal HCT116, and breast BT‐20 cells (Figure S1D,E, Supporting Information). We further leveraged the rapid pHe switch experiments to assess the impact of several well‐characterized FA transporter inhibitors, including those targeting CD36, FATP1, and FATP2. Notably, none of these inhibitors affected the increased capacity of DHA to promote LD formation under acidic conditions (Figure 1H; Figure S1F,G, Supporting Information). Interestingly, at physiological pHe, a significant reduction in DHA‐induced LD formation was observed following treatment with SSO (an irreversible CD36 inhibitor) and FATP1‐specific inhibitor (FATP1i) (Figure 1H; Figure S1F,G, Supporting Information), consistent with the established role of these transporters in facilitating FA uptake under neutral pHe. Collectively, these findings strongly suggest that under acidic pHe, the enhanced FA uptake occurs independently of canonical FA transport proteins. Also, it is worth to note that an acid‐induced increase in macropinocytosis could be excluded. Dextran‐TMR indeed failed to reveal a preferred uptake at pH 6.5 (vs pH 7.4) (Figure S1H–I, Supporting Information).

To exclude any contribution of long‐term pre‐adaptation of cancer cells to pHe 6.5 in the above LD experiments, we next performed a stepwise reduction in pHe using native cancer cells (as in panel A). We found that LD accumulation exhibits a sigmoidal pHe relationship in SiHa, HCT116, FaDu and BT‐20 cancer cells (Figure 1I,J; Figure S1J,K, Supporting Information). The observed preferential FA uptake (and consecutive accumulation for DHA) under rapid swap to an acidic pHe suggested a possible influence of the level of FA protonation. To address this hypothesis, we used the pH reporter protein (pHluorin2) to track the acidification of the intracellular compartment upon exposure of cancer cells to DHA. We found that pHe swapping to 6.5 led to cytosolic acidification but importantly that the presence of DHA in the extracellular medium further exacerbated this intracellular acidification contrary to what was observed in cells maintained at pHe 7.4 (Figure 1K,L). A time‐course study of DHA‐triggered acidification in cancer cells swapped to pHe 6.5 revealed that the region near the inner phospholipid layer was the first to show a change in intracellular pH (pHi) (Figure S1L, Supporting Information). Finally, to move beyond LD as the sole surrogate marker of enhanced DHA uptake under acidic pHe, we also examined its contribution to the phospholipid (PL) pool, as determined by LC‐MS. We observed a significant increase in DHA incorporation into PLs under acidic conditions compared to pHe 7.4 (Figure 1M). Notably, this pHe‐dependent effect was abolished when methyl ester–conjugated DHA was used in place of free DHA (Figure 1M), supporting the notion that the uncharged form of DHA more readily traverses the plasma membrane in an acidic microenvironment.

### 2.2 Extracellular Acidification by Hypoxia or Pharmacological Stimulation of Glycolysis Enhances DHA Uptake Across Cancer Cell Types

**Figure 2:**
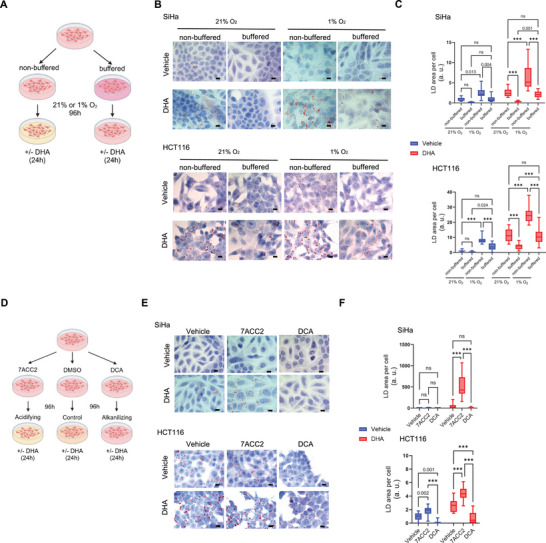
Extracellular acidification by hypoxia or stimulated glycolysis enhances DHA uptake across cancer cell types. A) Schematic protocol for inducing hypoxia (1% O_2_) in cancer cells cultured in either non‐buffered or buffered bicarbonate buffer, resulting in spontaneous medium acidification or preventing pHe drop, respectively. B,C) Representative pictures B) and quantification C) of ORO‐stained LD in SiHa and HCT116 cells supplemented with 25 µm DHA or FA‐free BSA as vehicle in the indicated conditions (N = 2, n = 10 fields). D) Schematic protocol for reducing or increasing pHe using MPC inhibitor 7ACC2, and PDK inhibitor DCA, respectively. E,F) Representative pictures E) and quantification F) of ORO‐stained LD in SiHa and HCT116 cells supplemented with 25 µm DHA or FA‐free BSA as vehicle after the indicated pharmacological treatments (DMSO as vehicle) (N = 2, n = 10 fields). All scale bars: 10 µm. The vehicle consists of an equivalent concentration of FA‐free BSA for DHA and DMSO for drugs. All graphs are presented as min‐max whisker plots, with the range indicated by Q1 to Q3, and the line representing the median. N = biological replicates, and n = technical replicates. ns = non‐significant; ***p < 0.001. Significance was determined by two‐way ANOVA with Tukey's multiple comparison test.

In the above sets of data, the pHe was controlled using HEPES/PIPES‐based buffer. To go deeper in the understanding of the pHe impact on FA uptake, we used hypoxia to mimic natural acidifying conditions. To distinguish the role of acidic pHe from hypoxia itself, we carried out FA uptake experiments in high bicarbonate buffer (**Figure** **2**A) to limit pHe dropping by the end of the experiments (vs low bicarbonate buffer) (Figure S2A, Supporting Information). We found that in response to 24 h DHA exposure, LD levels significantly increased under hypoxia and interestingly, LD accumulation was significantly lesser in the high bicarbonate buffer (i.e., in conditions where pHe drop was attenuated) (Figure 2B,C; Figure S2B,C, Supporting Information). This prominent role of the acidic component (vs reduced pO2 itself) in preferred FA uptake was observed across four distinct cancer cell lines, supporting a phenomenon that is not dependent on specific gene mutations.

In another set of experiments, we used two different drugs to modulate the pHe, namely 7ACC2, a MPC inhibitor known to prevent pyruvate transport into mitochondria and consecutively stimulate glycolysis,^[^ ^27^ ^]^ and DCA, a PDK inhibitor, used to further enhance pyruvate handling by the mitochondrial and reduce glycolytic turnover (Figure 2D).^[^ ^28^ ^]^ End‐point pHe measurements validated the opposite drug effects, i.e., a pHe reduction in response to 7ACC2 treatment and a pHe increase in response to DCA (Figure S2D, Supporting Information). Using this strategy, we found that LD accumulation (used as a read‐out of DHA uptake) was consistently increased in response to 7ACC2 and reduced upon DCA exposure in the four tested cell lines (Figure 2E,F; Figure S2E,F, Supporting Information). Importantly, we verified that the observed effects were not due to changes in the availability of glycerol‐3‐phosphate (G3P), the glycerol backbone required for esterifying fatty acids into triglycerides (TG). Both treatments resulted in a reduction in total G3P levels compared to vehicle, with no significant difference between the two inhibitors (Figure S2G, Supporting Information). The decrease in G3P observed in MPCi‐treated cells may reflect its consumption for TG synthesis. Still, the absence of LD formation without exogenous DHA supplementation (see blue bars in Figure 2F) indicates that increased fatty acid availability remains the primary driver of TG synthesis under acidic conditions. As for the reduction in G3P levels following DCA treatment, this likely reflects diminished glycolytic flux due to enhanced mitochondrial pyruvate oxidation. Although this could contribute to reduced TG synthesis, it is noteworthy that in HCT116 and FaDu cancer cells where LD formation is reduced by approximately 4‐ to 5‐fold, the G3P pool is only modestly decreased, by 25% and 10%, respectively (Figure 2F; Figure S2F,G, Supporting Information). Altogether, these data indicate that dynamic acidification (i.e., not stably imposed by buffering compounds) resulting from environmental hypoxic conditions or using a metabolic drug to enhance glycolytic turnover, favors an increase of FA uptake and subsequent storage.

### 2.3 Enhanced DHA Uptake Under Acidosis Sensitizes Cancer Cells to the Inhibition of Peroxisomal Activity

**Figure 3:**
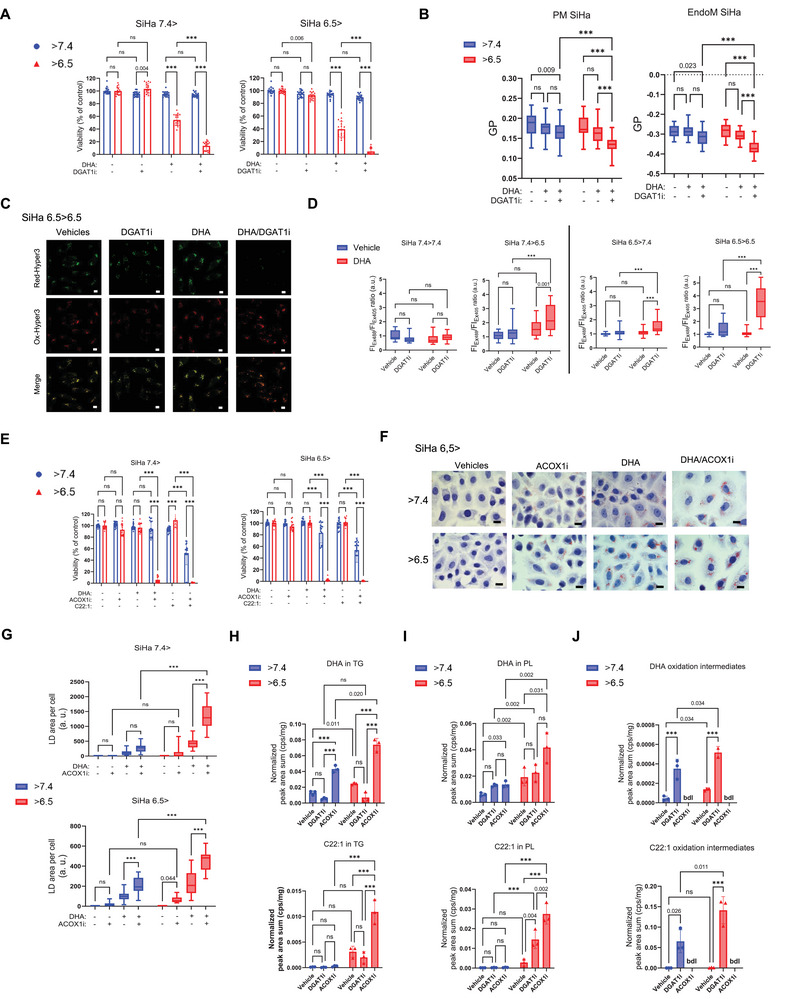
Enhanced DHA uptake under acidosis sensitizes cancer cells to peroxisomal inhibition. SiHa cells maintained at the indicated pHe (7.4 > 7.4 and 6.5 > 6.5) or following pHe swapping (7.4 > 6.5 and 6.5 > 7.4) were supplemented with 50 µm DHA or FA‐free BSA as vehicle, and treated as indicated. A) Effects of DGAT1 inhibition (15 µm A922500 vs DMSO, 72 h) in the different pHe conditions (N = 3, n = 6). B) Quantification of membrane fluidity in SiHa cells exposed for 48 h to 15 µm DGAT1i (or DMSO as vehicle); Generalized polarization (GP) was calculated for both plasma and endo‐membranes (n = 30 independent cells). C,D) Representative pictures of the Hyper3 oxidized and reduced forms in transduced SiHa cells maintained at acid pH 6.5 C) and quantification of Ex_488_/Ex_405_ Ratio (reflecting H_2_O_2_ production) normalised to control in SiHa cells exposed for 48 h to 15 µm DGAT1i (or DMSO as vehicle) in each pHe condition (N = 2, n = 10 fields) D). E) Effects of ACOX1 inhibition (60 µm tricosadiynoic acid vs DMSO, 24 h) in the different pHe conditions during supplementation with 50 µm DHA, C22:1, or FA‐free BSA as vehicle (N = 3, n = 6). F,G) Representative pictures F) and quantification G) of ORO‐stained LD in SiHa cells undergoing the indicated pHe changes and exposed to 30 µm ACOX1i for 48 h (N = 2, n = 10 fields). H–J) HPLC‐MS quantification of DHA or C22:1 esterified in TG H) and in Phospholipids I), or their β‐oxidation intermediates J) from SiHa cells maintained at 7.4 (7.4 > 7.4) or shifted to 6.5 (7.4 > 6.5) and supplemented with 50 µm DHA or C22:1 and treated with 7.5 µm DGAT1i or 15 µm ACOX1i (DMSO as vehicle) during 24 h (N = 3, n = 1). All scale bars: 10 µm. The vehicle consists of an equivalent concentration of FA‐free BSA for DHA or DMSO for drugs. Graphs are presented as mean ± SD A,E,H,I,J) or min to max whisker plots, range = Q1 to Q3, and line = median B,D,G). bdl = below detection limit. N = biological replicates, and n = technical replicates. ns = non‐significant; ***p < 0.001. Significance was determined by two‐way ANOVA with Tukey's multiple comparison test.

While LD formation under acidosis was thought to result from a pre‐existing genetic or transcriptomic rewiring of FA metabolism in cancer cells,^[^ ^29^, ^30^ ^]^ the current study instead points toward an upstream critical contribution of a preferred FA uptake regardless of cell phenotypic adaptations. We therefore next examined the effects of an inhibitor of diacylglycerol acyl transferase (DGAT), the rate‐limiting enzyme in TG synthesis, in cancer cells undergoing acute pHe swapping experiments (instead of cancer cells chronically adapted to pHe 6.5 as previously reported).^[^ ^26^ ^]^ We found that the pHe 7.4 > 6.5 shift at the time of DHA and DGATi exposure induced dramatic cytotoxic effects while the acute pHe 6.5 > 7.4 shift prevented the development of cell toxicity (**Figure** **3**A; Figure S3A, Supporting Information). In the same sets of experiments, cells maintained at acidic pHe (6.5 > 6.5) were sensitive to the DHA/DGATi combination while cells at physiological pHe (7.4 > 7.4) remained insensitive to it (Figure 3A; Figure S3A, Supporting Information). Of note, we found that addition of DHA at the time of the pHe 7.4 > 6.5 shift led to a reduction in pHi independently of the presence of DGATi (Figure S3B, Supporting Information).

Since LD formation is a major way to handle excess FA, in particular peroxidable DHA,^[^ ^26^ ^]^ we hypothesized that inhibition of LD biogenesis should alter the fate of DHA and possibly overload other FA‐fueled metabolic pathways. To evaluate a possible redirection of DHA into phospholipids, we first used the ratiometric fluorescent dye Laurdan to track the fluidity state of cell membranes undergoing the above acute pHe shift. We calculated the generalized polarization (GP) parameter which is inversely proportional to membrane fluidity,^[^ ^31^ ^]^ after excluding the contribution of Laurdan‐labelled lipid droplets to the fluorescent signal. We found that combining DHA with DGAT1i increased the fluidity of plasma and endo‐membranes (lower values of GP), an effect accentuated at acidic pHe (Figure 3B; Figure S3C, Supporting Information).

Besides the enrichment of DHA in membranes, we examined whether an enhanced DHA metabolization could be detected in acid‐exposed cancer cells. For a very long chain PUFA as DHA, peroxisomal β‐oxidation is known to represent the main path of degradation.^[^ ^32^, ^33^ ^]^ To track potential changes in peroxisomal activity, we used a ratiometric fluorescent H_2_O_2_‐sensitive protein, targeting the peroxisome thanks to a PTS1 sequence (HyPer3‐PTS1).^[^ ^34^ ^]^ We first confirmed the colocalization of the HyPer3‐PTS1 reporter protein and ABCD3, a specific peroxisomal protein (Figure S3D, Supporting Information) and the capacity to discriminate reduced and oxidized Hyper3 probe in the presence of DHA and/or DGATi (Figure 3C). Then, we quantified the production of peroxisomal H_2_O_2_ in the different pHe switch conditions detailed above. Experiments performed using cancer cells undergoing pHe 7.4 > 6.5 swap revealed a significant increase in peroxisomal H_2_O_2_ in the combined presence of DHA and DGAT1i but not when only DHA was added (Figure 3D; Figure S3E,F, Supporting Information). This result suggests that inhibiting excess DHA buffering by LDs leads to an increase in peroxisomal β‐oxidation rate. Similar results were obtained when acid‐adapted cancer cells were maintained under acidic pHe (pH 6.5 > 6.5) (see Figure 3C) while the acute pHe 6.5 > 7.4 swap largely reduced the capacity of DHA/DGAT1i to promote H_2_O_2_ production (Figure 3D; Figure S3E,F, Supporting Information); no significant changes in H_2_O_2_ production were observed in the pHe 7.4 > 7.4 condition whatever the treatment (Figure 3D; Figure S3E,F, Supporting Information). To further substantiate the enhanced involvement of peroxisomal FA metabolism under acidic pHe, we used the very‐long‐chain C22:1 monounsaturated FA (MUFA), a prototypical substrate handled by peroxisomes. Inhibition of LD formation in the presence of C22:1 resulted in a significant increase in H_2_O_2_ production under acidic pHe, but not at pHe 7.4 (Figure S3G,H, Supporting Information).

We next directly investigated the contribution of peroxisomal activity in the handling of enhanced DHA uptake under acidic conditions. For this purpose, we targeted ACOX1, the rate‐limiting enzyme of peroxisomal β‐oxidation. We found that cells maintained at acidic pHe at the time of treatments (6.5 > 6.5) as well as cells acutely swapped from physiological to acidic pHe (7.4 > 6.5) were particularly sensitive to the combination of either DHA or C22:1 and the ACOX1 inhibitor 10,12‐tricosadiynoic acid^[^ ^35^ ^]^ (Figure 3E; Figure S3I, Supporting Information). These effects were in line with the significant reduction in H_2_O_2_ production measured using the HyPer3‐PTS1 probe^[^ ^34^ ^]^ (Figure S3J,K, Supporting Information). Strikingly, cells maintained at physiological pHe (7.4 > 7.4) but also cells acutely shifted from acidic to physiological pHe (6.5 > 7.4) did not show reduction in cell viability when exposed to DHA in the presence of ACOXi (Figure 3E; Figure S3I, Supporting Information); peroxisomal H_2_O_2_ production was consistently unchanged in these two later conditions (Figure S3J,K, Supporting Information). Of note, combination of ACOXi and C22:1 resulted in significant cytotoxicity even at physiological pHe (in both 7.4 > 7.4 and 6.5 > 7.4 conditions), though to a lesser extent than under acidic conditions, where no viable cells remained (Figure 3E; Figure S3I, Supporting Information).

Given the FA‐like structure of the ACOX1 inhibitor, 10,12‐tricosadiynoic acid, which could potentially enhance drug uptake under acidic conditions, we additionally performed genetic silencing of ACOX1 using three distinct shRNAs (Figure S3L, Supporting Information). This approach yielded comparable results, with DHA supplementation inducing cytotoxicity in ACOX1 knockdown cells exclusively under acidic conditions (Figure S3M, Supporting Information).

Subsequently, we investigated whether ACOX1 inhibition itself could redirect PUFA toward LDs. For this purpose, a lower concentration of ACOX1i was employed, still sufficient to induce cell toxicity under acidic conditions (Figure S3N, Supporting Information). We observed a significant DHA‐dependent increase in LD formation across the four cancer cell lines treated with ACOX1i under the 6.5 > 6.5 and 7.4 > 6.5 experimental conditions (Figure 3F,G; Figure S3O,P, Supporting Information). Consistent results were obtained in ACOX1 knockdown cells, which exhibited a pronounced increase in LD formation upon DHA supplementation (Figure S3Q,R, Supporting Information). LC‐MS analysis also confirmed a net increase in the incorporation of DHA as well as C22:1 into TG, with significantly greater enrichment under acidic conditions (than at pH 7.4); DGAT inhibition instead led to a marked reduction in TG formation (Figure 3H). Interestingly, lipidomic profiling also revealed that ACOX1 inhibition significantly increased the incorporation of both DHA (Figure 3I; Figure S3S, Supporting Information) and C22:1 (Figure 3I) into phospholipids, while concurrently reducing the levels of oxidized intermediates derived from these VLCFAs (Figure 3J). Conversely, DGAT inhibition resulted in a substantial increase in the accumulation of oxidized byproducts (Figure 3J).

Overall, the preferential acidosis‐driven DHA and C22:1 uptake leads to both the stimulation of FA storage in lipid droplets and the peroxisomal oxidation, two pathways that operate like interconnected vessels, where inhibition of one stimulates the other. Importantly, when cells become overloaded, neither escape route (i.e., storage or oxidation) can prevent toxicity.

### 2.4 DHA and ACOX1i Combination Induces ROS Production and ER Stress, Leading to Apoptosis Without Triggering Ferroptosis

**Figure 4:**
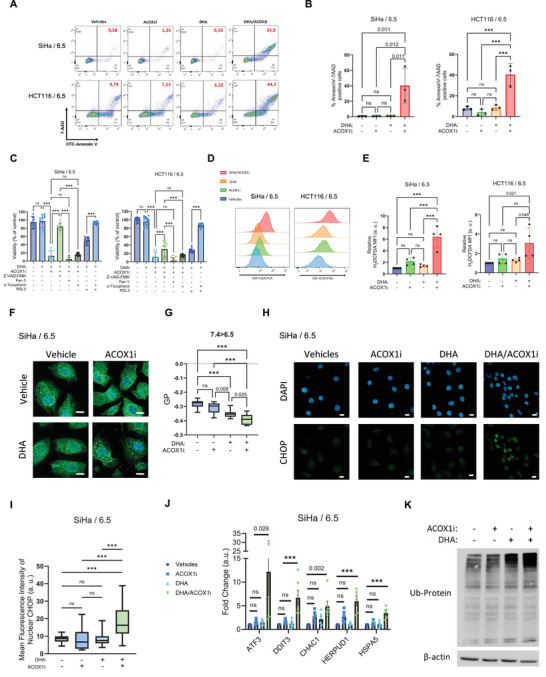
DHA and ACOX1i combination induce apoptosis without triggering ferroptosis. SiHa and HCT116 cells maintained at acidic pHe 6.5 were supplemented with 50 µm DHA or FA‐free BSA as vehicle, and treated as indicated. A) Representative flow cytometry plots depicting 7‐AAD versus FITC‐Annexin V staining of pH6.5‐adapted SiHa and HCT116 cells treated for 8 h with 60 µm ACOX1i (DMSO as vehicle). B) Quantification of the cell number (%) in the 7‐AAD and Annexin V double‐positive quadrants (N = 3, n = 1). C) Effect of cell death inhibitors (30 µm Z‐VAD‐FMK for apoptosis or 30 µm Ferrostatin‐1 and 20 µm α‐tocopherol for ferroptosis) on the viability of SiHa and HCT116 cells exposed to 40 µM ACOX1i, or 5 µm RSL3 as positive control of ferroptosis (DMSO as vehicle) (N = 3, n = 6). D,E) Representative flow cytometry histograms D) and quantification E) of ROS in SiHa and HCT116 cells treated for 5 h with 60 µm ACOX1i (DMSO as vehicle), as determined using DCFDA (N = 4, n = 1). F) Live cell confocal microscopy assessment of ER morphology in SiHa upon exposure for 5 h to 60 µm ACOX1i (DMSO as vehicle), as determined using 1 µm ER‐tracker green reporter. G) Quantification of membrane fluidity (2 µm Laurdan) in SiHa cells exposed for 24 h to 15 µm ACOX1i (or DMSO as vehicle); Generalized polarization (GP) was calculated for ER membrane thanks to 1 µm ER‐tracker red staining (N = 3, n = 4). H,I) Representative pictures H) and quantification I) of nuclear CHOP immunofluorescence in fixed SiHa cells treated for 4 h with 60 µm ACOX1i (DMSO as vehicle) (n = 60 independent cells). J,K) RT‐qPCR of genes associated with UPR J) and immunoblotting of ubiquitinylated proteins K) in SiHa cells treated for 5 h with 60 µm ACOX1i (DMSO as vehicle) and 50 µm DHA treatments (FA‐free BSA as vehicle) (N = 6 for qPCR and N = 1 for Immunoblotting). All scale bars: 10 µm. Graphs are presented as mean ± SD B,C,E), mean ± SEM J), or min to max whisker plots, range = Q1 to Q3, and line = median G,I). N = biological replicates, and n = technical replicates. ns = non‐significant; ***p < 0.001. Significance was determined by one‐way ANOVA B,E,G,I,J) and two‐way ANOVA C) with Tukey's multiple comparison test.

To further support the notion that ACOX1 dependency constitutes a metabolic vulnerability in acid‐exposed cancer cells with enhanced uptake of VLCFAs (e.g., DHA and C22:1), we performed experiments using the shorter MUFA oleate (C18:1), which can be metabolized by mitochondria in the absence of peroxisomal activity. Accordingly, cancer cells maintained at pHe 6.5 and exposed to oleate did not exhibit the same pronounced loss of viability observed with DHA or C22:1 (Figure S4A, Supporting Information). We next examined the type of cell death occurring in acidic cancer cells and found that ACOX1 inhibition in the presence of DHA led to a net increase in double‐positive 7‐AAD/AnnexinV cells with an apparent transition through the 7‐ADD‐negative Annexin V‐positive quadrant, supporting apoptosis as the main cell death pathway (**Figure** **4**A,B). This observation differed from previous work identifying ferroptosis as the cell death mechanism for acidic cancer cells exposed to DHA and DGAT1i,^[^ ^26^ ^]^ yet remained compatible with it. Indeed, as revealed by LC‐MS experiments, DGAT inhibition prevented sequestration of excess DHA into TG whereas ACOX inhibition led to its preferred incorporation in TG and thus into LD (Figure 3H), rendering them less susceptible to peroxidation. To explicitly address the nature of cell death in response to ACOX inhibition, we next used Z‐VAD‐FMK and Ferrostatin‐1, inhibitors of apoptosis and ferroptosis, respectively, and found that only the apoptosis inhibitor partially rescued the viability of the different cancer cell lines exposed to DHA/ACOX1i (Figure 4C; Figure S4B, Supporting Information). Notably, ferroptosis inhibitor α‐tocopherol also failed to prevent DHA/ACOXi toxicity under conditions where the effects of canonical ferroptosis inducer RSL3 were completely blunted (Figure 4C; Figure S4B, Supporting Information), confirming the non‐ferroptotic mode of cell death. Also, co‐treatment with ACOXi and DGATi produced an additive cytotoxic effect that was only minimally, or not at all, reversed by α‐tocopherol, further supporting a predominant role for apoptosis in ACOX1i‐induced cytotoxicity despite the ferroptosis‐sensitizing action of DHA (Figure S4C, Supporting Information).

Next, we examined whether increased reactive oxygen species (ROS) production could drive apoptosis in response to DHA and/or ACOX1i. DCFDA measurements revealed that ROS levels were indeed increased when DHA and ACOX1i were combined but not when added separately (Figure 4D,E). Interestingly, measurements of mitochondrial ROS production using Mitosox failed to detect any change suggesting that modulation of the respiratory complex chain by the combination of ACOX1i and DHA was unlikely to play a major role in the observed oxidative stress (Figure S4D,E, Supporting Information). Since the peroxisomal H_2_O_2_ production was also reduced in these conditions (Figure S3J,K, Supporting Information), we looked whether the increase in DCFDA‐sensitive ROS could lead to apoptosis through induction of ER stress. We found that the DHA/ACOX1i combo treatment was associated with (i) the acquisition of a punctate ER morphology as previously reported in cells undergoing unfolded protein response (UPR)^[^ ^36^ ^]^ (Figure 4F; Figure S4F, Supporting Information), (ii) an increase in the fluidity of the ER membranes (ie, reduction in GP) as determined by combining fluorescent Laurdan and ER‐tracker dye detection (Figure 4G; Figure S4G, Supporting Information) and (iii) a highly significant increase in the expression of CHOP, a key transcription factor activated during ER stress and mediating apoptosis (Figure 4H,I; Figure S4H,I, Supporting Information). Western blot analyses also revealed a ACOX1i/DHA‐induced increase in phospho‐p38 MAPK, phospho‐EIF2α and phospho‐IRE1α, that are signals commonly associated with ER stress, independently of the cancer cell types (Figure S4J,K, Supporting Information). qPCR analysis confirmed upregulation of additional ER stress markers in cancer cells treated with ACOXi and DHA (Figure 4J; Figure S4L, Supporting Information). This was accompanied by an accumulation of ubiquitinated proteins (Figure 4K; Figure S4M, Supporting Information), consistent with proteasomal overload due to increased protein misfolding.

### 2.5 Acidosis‐Inducing MPCi Enhances the Anticancer Effects Resulting from Peroxisomal Activity Saturation

**Figure 5:**
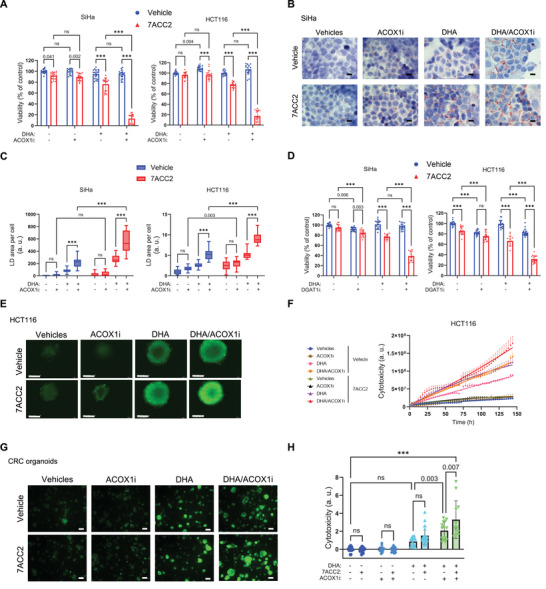
Acidosis‐inducing MPCi enhances the cytotoxic effects of DHA supplementation. Cancer cells, spheroids, and organoids were pre‐challenged with 10 µM 7ACC2 (DMSO as vehicle) to stimulate glycolysis and induce medium acidification and treated as indicated with ACOX1 inhibitor (tricosadiynoic acid) (DMSO as vehicle) and DHA (FA‐free BSA as vehicle). A) Effects of combining 75 µm DHA and ACOX1 inhibition (60 µm, 72 h) on the viability of SiHa and HCT116 cells under induced acidification (N = 3, n = 6). B,C) Representative pictures B) and quantification C) of ORO‐stained LD in SiHa and HCT116 cells under induced acidification and exposed to 50 µm DHA and 30 µm ACOX1i (N = 2, n = 10 fields). Scale bar: 10 µm. D) Effects of combining 75 µm DHA and DGAT1 inhibition (30 µm, 72 h) (DMSO as vehicle) on the viability of SiHa and HCT116 cells under induced acidification (N = 3, n = 6). E,F) Representative pictures E) and quantification F) of cytotoxic effects measured by Cytotox Green Reagent of combining 100 µm DHA and 60 µm ACOX1i in HCT116 spheroids under induced acidification (N = 3). Scale bar: 300 µm. G,H) Representative pictures G) and quantification H) of cytotoxic effects of combining 50 µm DHA and 60 µm ACOX1i on colorectal cancer organoids under induced acidification (N = 18). Scale bar: 100 µm. Graphs are presented as mean ± SD A,D, H), mean ± SEM F), or min to max whisker plots, range = Q1 to Q3, and line = median C). N = biological replicates, and n = technical replicates. ns = non‐significant; ***p < 0.001. Significance was determined by two‐way ANOVA with Tukey's multiple comparison test.

While we provide compelling evidence that the combination of DHA with ACOX1i may be particularly relevant to kill cancer cells residing in the acidic tumor compartment, the failure of the combo to kill cancer cells at pHe 7.4 indicates that a large proportion of cancer cells are likely to escape such therapeutic approach. In the next series of experiments, we therefore evaluated the acidifying capacity of MPC inhibitor to sensitize cancer cells to the effects of the ACOX1i/DHA combination. We found that, independently of the cancer cell type, ACOX1i enhanced the detrimental effects of DHA more efficiently in cancer cells pre‐challenged with MPCi, 7ACC2 (**Figure** **5**A; Figure S5A, Supporting Information). As additional evidence that MPC inhibition contributed to a net increase in DHA uptake, we identified a parallel increase in LD formation when cancer cells were exposed to DHA and ACOX1i (Figure 5B,C; Figure S5B,C, Supporting Information). Similarly, we showed that inhibiting LD formation by DGATi induced more profound cytotoxic effects when cancer cells were pre‐treated with MPCi (Figure 5D).

Next, we used tumor spheroids and patient‐derived tumor organoids (PDTOs) to recapitulate situations where cancer cells face a spontaneous acidification of the extracellular medium and addition of MPCi to extend the proportion of cells undergoing acidosis. Here, we preferentially used HCT116 and FaDu cancer cells to generate spheroids considering the lesser ability to do so for SiHa and BT20 cells. Cytotoxicity was tracked using IncuCyte device in 3D spheroids or patient‐derived colorectal cancer organoids pre‐treated for 96 h with MPCi 7ACC2 and then exposed to DHA with or without ACOX1i; medium acidification was validated in response to 7ACC2 exposure in spheroids (see Figure 2D) and PDTOs (Figure S5D, Supporting Information). We identified a significant detrimental effect of DHA supplementation on MPCi‐treated spheroids (Figure 5E,F; Figure S5E,F, Supporting Information) and PDTOs (Figure 5G,H), an effect further accentuated in the presence of ACOX1i (Figure 5E–H; Figure S5E,F, Supporting Information).

Altogether, these data demonstrate that MPC inhibition, by its capacity to promote acidification of the extracellular milieu bathing 3D spheroids and PDTOs, has the potential to sensitize cancer cells exposed to a DHA and ACOX1i combination.

### 2.6 Mouse and Human Dietary ω3‐PUFA Supplementation Enhances ACOX1 Toxicity in Animal Tumors and Patient‐Derived Organoids in an Acid‐Dependent Manner

**Figure 6:**
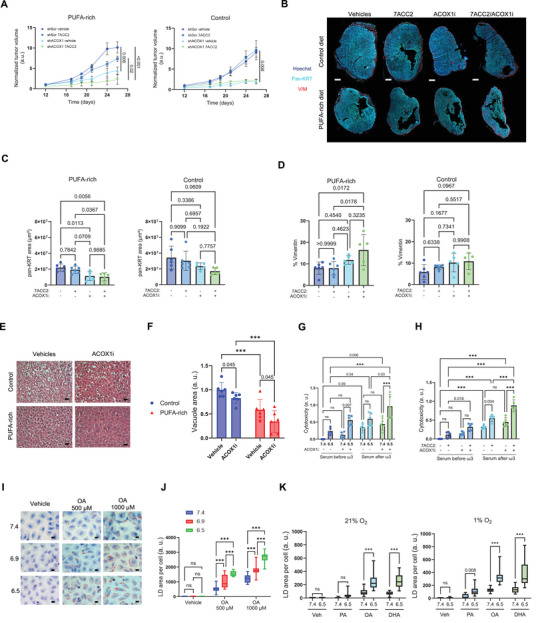
Acidosis enhances the detrimental effects of combining dietary ω3‐PUFA and ACOX1 and promotes FA uptake in non‐cancer cells. A) Tumor growth in mice injected with SiHa cancer cells transduced with ACOX1 shRNA (vs scramble shRNA). Mice were fed with control (left) or PUFA‐rich (right) diets and treated or not with MPCi 7ACC2 (3 mg kg^−1^) (N = 5). B) Representative pictures of FaDu tumors collected from mice fed either diet and treated with ACOX1i (10 mg kg^−1^), MPCI 7ACC2 (3 mg kg^−1^), or both. Scale bar: 1 mm. C,D) Quantification of cancer cells detected by pan‐keratin staining C) and fibroblasts detected by vimentin staining D) in FaDu tumors collected from mice fed either diet, and treated as in B (N = 5,6). E,F) Representative pictures of liver sections from mice bearing FaDu tumors and treated as in B (E) and quantification of the extent of liver toxicity revealed by vacuolization (F) (N = 6). Scale bar: 30 µm. G,H) Cytotoxic effects of 60 µm ACOX1i (DMSO as vehicle) on human colorectal cancer organoids cultured at pHe 7.4 or 6.5 G) or undergoing medium acidification using 7ACC2 treatment (DMSO as vehicle) H), and supplemented with human serum from 5 healthy donors, before and after 1‐week ω3 PUFA dietary supplementation (N = 5, n = 4). I,J) Representative pictures I) and quantification J) of ORO‐stained LD in endothelial cells cultured at the indicated pHe, and supplemented with 500 or 1000 µm OA (FA‐free BSA as vehicle) (N = 3, n = 4 fields). Scale bar: 10 µm. K) Quantification of ORO‐stained LD in cardiomyocytes cultured at pHe 7.4 or 6.5, either under normoxia or hypoxia (1% O_2_), and supplemented with 50 µm PA, OA, or DHA (FA‐free BSA as vehicle) (N = 3, n = 4 fields). Graphs are presented as mean ± SD C,D,F,G, and H), mean ± SEM A), or min to max whisker plots, range = Q1 to Q3, and line = median J and K). N = biological replicates and n = technical replicates. ns = non‐significant; ***p < 0.001. Significance was determined by one‐way ANOVA, Tukey's multiple comparison test (A with endpoints, C, D), and two‐way ANOVA, Tukey's multiple comparison test F,G,H,J,K).

To explore in vivo the pro‐acidification strategy to sensitize cancer cells to PUFA, we fed mice either with control diet or isocaloric ω3 PUFA‐rich diet, and after two weeks of diet habituation, we injected SiHa cancer cells knockdown for ACOX1 (vs control SiHa cells). When tumors from control condition reached 35 mm^3^, mice were treated with 3 mg.kg^−1^ MPCi, 7ACC2, and the tumor volume was followed over time; we had previously shown using CEST‐MRI with iopamidol as a pHe probe that MPC inhibition significantly reduces tumor pHe.^[^ ^37^ ^]^ Independently of the diet, the silencing of ACOX1 drastically affected tumor development (**Figure** **6**A), supporting a major role of ACOX1 in growing tumors. We therefore conducted a similar in vivo experiment with ACOX1i administered in mice bearing already developed tumors and pre‐treated (or not) for one week with 3 mg.kg^−1^ 7ACC2. We did not observe any significant effects of the combination of 7ACC2 with ACOX1i (neither of each drug administered alone) in mice fed either diet (not shown). However, when tumors were collected, we observed a distinct morphological appearance dependent on the diet and the treatments, together with the release of an abundant liquid exudate when mice were fed the ω3‐rich diet. Since this liquid accumulation may have led to an underestimation of changes in tumor mass when measured with a calliper, we carried out a careful analysis of paraffin‐embedded tumors through multiplex immunolabeling (Figure 6B). This histological analysis confirmed that tumors from mice fed the ω3 PUFA‐rich diet consistently exhibited a smaller cell density (see overall tumor sections in Figure 6B) and would tear more easily upon sectioning, supporting a larger extent of cell death. Quantification of non‐necrotic cancer cells in tumor sections using a pan‐keratin antibody confirmed that ACOX1 inhibition significantly reduced tumor growth in mice fed the ω3 PUFA‐rich diet, but not in those on the control diet (Figure 6C). Co‐treatment with 7ACC2 did not further enhance the growth inhibitory effects of ACOX1i, although a trend toward increased vimentin‐positive stromal cells was observed, suggesting fibroblast colonization of the areas vacated by dying cancer cells (Figure 6D). In parallel, to assess potential deleterious effects of the ACOX1i treatment on overall mouse health, we examined hepatic tissue integrity. Mice fed the ω3‐rich diet exhibited reduced hepatic steatosis compared to those on the control diet. Interestingly, ACOX1 inhibition further improved liver health, with this effect being particularly pronounced in mice receiving the ω3‐rich diet (Figure 6E,F).

Finally, we designed a preliminary clinical study using human patient‐derived tumor organoids (PDTOs) and sera of human volunteers collected before and after one week of fish oil‐derived ω3 PUFA supplementation (**Table** **1**).

**Table 1:** Demographic and dietary information of study volunteers.

Volunteer	A	B	C	D	E
Age	27	26	27	28	26
Sex	M	F	F	M	F
Weight (kg)	82	64	60	70	54
Height (cm)	187	163	169	184	163
BMI	23,4	24,1	21	20,7	20,3
Fish consumption frequency	< than once a week	< than once a week	Once a week	< than once a week	Never
ω3 enriched products consumption frequency	< than once a week	Never	Never	Never	Never

Lipid analysis through GC showed an increase in the serum content of ω3 PUFAs (ie, EPA and DHA) in all volunteers (Figure S6A,B, Supporting Information). While PDTOs can grow ex vivo, they rarely reach size compatible with the spontaneous development of hypoxia and acidosis. We therefore used PDTOs cultured under physiological conditions (pHe 7.4) but also in a medium buffered at pHe 6.5 to recapitulate deeper acidic conditions encountered in vivo. Exposure for one week to 60 µM ACOX1i revealed that at both pHe, post‐ω3 PUFA supplementation serum led to an increased cell toxicity (vs pre‐supplementation control serum) (Figure 6G). Notably, acidic pHe further amplified the cytotoxic effects of ACOX1i in cells exposed to ω3 PUFA‐supplemented serum (Figure 6G; Figure S6C, Supporting Information). We next used MPCi 7ACC2 as a pretreatment to induce acidosis, rather than artificially buffering the PDTO culture medium at pHe 6.5. This approach revealed that ACOX1i and MPCi exerted additive cytotoxic effects in PDTOs exposed to serum collected post‐ω3 PUFA supplementation (Figure 6H).

### 2.7 Uptake of FA is Enhanced in Cardiovascular Tissues Experiencing Local Extracellular Acidosis

At the end of our tumor‐related study, we reasoned that the preferential uptake of FA when the extracellular milieu becomes acidic should not only apply to cancer cells but any tissues experiencing local acidification. Such reduction in pHe is actually reported in cardiovascular diseases, in particular atherosclerosis^[^ ^38^ ^]^ and cardiac ischemia.^[^ ^39^ ^]^ We therefore exposed endothelial cells and cardiac myocytes to an acidic medium and analysed the fate of exogenous FA. We found that LD accumulation in endothelial cells exposed to oleic acid (OA), the most abundant circulating FA, was enhanced in proportion to the reduction in pHe (Figure 6I,J). Similarly, rat neonatal cardiomyocytes exposed to PA, OA or DHA accumulated more LD when cultured at pHe 6.5 (vs pHe 7.4) or under hypoxia (and associated increased glycolysis) (Figure 6K; Figure S6D, Supporting Information). Interestingly, PA only accumulated into LD under hypoxia, suggesting that in the presence of oxygen, excess PA is consumed by the mitochondria.

## 3 Discussion

This study reveals that a primary driver of the cell metabolic adaptation to ambient acidosis is the transition of fatty acids from their saline to acidic form, excluding the widely assumed acid‐induced transcriptomic reprogramming that promotes a metabolic shift toward reliance on exogenous lipids. While the pH‐dependent ionization of (fatty) acid is a well‐known principle of chemistry, our work demonstrates the significance of this acid‐base paradigm within the biological context of tissue acidosis, as exemplified in cancer cells, endothelial cells and cardiac myocytes. In cancer, the universality of this paradigm is further reinforced by consistent observations in acid‐exposed cancer cells from diverse origins (e.g., cervix, colorectal, head and neck and breast), demonstrating its independence from genetic background. Proton accumulation in tumors results from both a deficit in vascular wash‐out and an excess production largely resulting from anaerobic and aerobic glycolysis.^[^ ^19^, ^20^ ^]^ While glycolysis and acidosis are commonly identified as pharmacological targets in cancer therapy, our study demonstrates that enhancing glycolysis and, in turn, exacerbating acidosis creates a unique opportunity to poison cancer cells with excess fatty acids. We found that this acid‐evoked vulnerability of cancer cells can be exploited by very long‐chain PUFA, in particular the omega‐3 FA DHA, owing to their reliance on peroxisomes for metabolism. This essential peroxisomal processing, which generates shorter fatty acyl‐CoA for mitochondrial processing, also explains why PUFA that are not readily processed tend to accumulate as TGs within LDs (vs PA – see Figure 1C). Here, we showed that inhibiting the peroxisomal enzyme ACOX1 may actually overload the capacity of LD formation to act as a salvage pathway for acid‐exposed cancer cells. Remarkably, while PUFA‐dependent cell death usually refers to ferroptosis resulting from peroxidation, we found instead that unresolved ER stress resulting from ACOX1 inhibition leads to apoptosis in the presence of PUFA. In the presence of ACOX1i, the excess PUFA entering the cell eventually ends up overwhelming the ER, where FA are normally incorporated into triglycerides and phospholipids.^[^ ^30^, ^40^ ^]^ Consequences are enhanced oxidative stress in the cytosol and alterations in normal protein folding leading to UPR. Prolongation of this ER stress then activates pro‐apoptotic signaling pathways through proteins such as CHOP (C/EBP homologous protein), which leads to cell death (Figure 4G,H).

We used rapid pHe swapping to demonstrate that metabolic gene reprogramming does not drive the preferential uptake of FAs in cells suddenly exposed to extracellular acidification, nor the reverse reduction in FA uptake when acidic pHe is acutely restored to a physiological level. To capture these rapid changes, we employed both radiolabeled FAs and holotomography (label‐free, time‐lapse imaging of LD dynamics), which revealed a minute‐range enhancement of FA uptake in acid‐exposed cancer cells. Moreover, we showed that while FA transporter inhibitors can block uptake at physiological pHe, the burst of FA uptake under acidic conditions is unaffected by transporter inhibition. We also observed that hypoxia‐related acidosis promoted preferential FA uptake, an effect that could be dissociated from hypoxia‐induced gene reprogramming through pHe clamping. In addition, we showed that drugs able to alter pHe through stimulation or inhibition of glycolysis (i.e., via MPC blockade or PDK inhibition) could reinforce or prevent FA uptake, respectively. This demonstration presents key implications for the therapeutic exploitation of enhanced PUFA capture by acid‐exposed cancer cells. First, it indicates that more a tumor is acidotic (ie, more it contains acidic areas), more it will respond to PUFA supplementation and ACOX1 inhibition. Second, the pharmacological enhancement of glycolytic flux induced by MPCi administration may considerably amplify the effects of DHA supplementation and/or ACOX1i. We validated this statement in cancer cells, and importantly in more clinically relevant spheroid models and patient‐derived tumor organoids. While we previously reported that MPC inhibition significantly reduces tumor pHe in mice,^[^ ^37^ ^]^ its effect in the present in vivo model was marginal, if any, suggesting that acidosis was already well established in the tumors used in this study.

As an indication of the clinical transferability of our approach, we further demonstrated that ACOX1 inhibition could benefit from the adjuvant anticancer effects of either dietary source of PUFA in mice or PUFA capsule supplementation in volunteers. In mice fed a diet enriched in PUFAs, we observed a dramatic decrease in cancer cell survival in response to MPCi and/or ACOX1i (compared to an isocaloric control diet). IHC analysis revealed a significant overall reduction in cancer cell count, with a large necrotic area likely contributing to increased fluid accumulation in the treated tumors (thereby obscuring the reduction in size measured by calipers and causing tissue tearing during sectioning). In human volunteers, we found that a 3‐fold enrichment in serum ω3‐PUFA could be achieved with a supplementation of 1 g DHA three times per day for one week. While the observed adjuvant antitumor effects are likely to result from the unconjugated form of ω‐3 PUFA, we cannot exclude that a lipase activity at the surface of cancer cells further contributed to the enhanced bioavailability of free PUFA.^[^ ^41^, ^42^ ^]^ Still, these data showed that reaching PUFA levels compatible with an enhanced toxicity of ACOX1i represents an achievable clinical goal. Furthermore, it should be emphasized that even in the presence of a large excess PUFA, peroxisome inhibition did not induce liver toxicity and was even protective confirming previous studies where liver‐specific ACOX1 KO reduced steatohepatitis and liver fibrosis through stimulation of lipophagy.^[^ ^34^, ^43^, ^44^ ^]^

## 4 Conclusion 

In conclusion, our study unveils a paradigm‐shifting perspective on lipid metabolism, where the microenvironment dictates the preference for FAs, which then steer the cell phenotype, rather than the reverse. This insight reveals a novel and highly druggable vulnerability in cancer but also opens exciting new avenues for targeted therapies in ischemic cardiovascular diseases.

## 5 Experimental Section

### Cancer Cells

Human cancer cell lines (HCT116, FaDu, SiHa, and BT‐20) were purchased from ATCC. They were stored according to the supplier's instructions and used within 6 months after resuscitation of frozen aliquots. Cell lines were cultured in home‐made medium, DMEM (#D5030, Merck) supplemented with 10% heat‐inactivated FBS (F7524, Merck), 10 mM D‐glucose (#G8272, Merck), 2 mM L‐Glutamine (#25030‐024, Gibco), 1% penicillin/streptomycin (P/S, #15140‐122, Gibco), 0.015‰ Phenol red (#P0290, Merck) and 25 mM of both HEPES (#H3375, Merck) and PIPES (#P2949, Merck). pH was adjusted to 7.4 and 6.5 by the addition of NaOH, and acidosis pH‐adapted tumor cells were established as previously described.^[^ ^22^ ^]^ Cell lines were regularly tested for mycoplasma contamination with the MycoAlert Mycoplasma Detection kit (LT07‐318, Lonza).

### Endothelial Cells

Bovine aortic endothelial cells (BAECs) were purchased from Cell Application Inc. (Cat# B304‐05). BAECs were maintained in culture plates pre‐coated with 0.2% gelatin (Cat# G1393, Sigma) and supplemented with Endothelial Cell Growth Medium (B211‐500, Cell Application Inc.). Following the supplier's guidelines, BAEC were passaged when they reached ≈80–90% confluence and were utilized up to the eighth passage.

### Cardiomyocytes

Cardiomyocytes (CMs) were isolated from the ventricular tissue of 3‐day‐old neonatal rats in a procedure approved by the ethics committee of UCLouvain and in agreement with national care regulations. Briefly, the heart was excised and immersed in cold HBSS (HBSS‐2A, Capricorn Scientific) before being dissected into smaller pieces. The CMs were then incubated in a flask containing a sterile trypsin‐HBSS solution (15 mg 25 mL^−1^, Trypsin; T4799‐5G, Sigma‐Aldrich) along with 1% P/S (15140‐122, Gibco) and 1% Amphotericin B (15290‐026, ThermoFisher) overnight at 4 °C with continuous agitation. Following this incubation, the trypsin‐HBSS solution was removed and replaced with complete DMEM medium (61965‐026, ThermoFisher) supplemented with 10% FBS (R‐6404352, Sigma‐Aldrich), 1% P/S, 1% Amphotericin B and 0.5% HEPES Buffer Solution (15630‐056 ThermoFisher) at 37 °C for five min. Subsequently, a digestion process using collagenase type II diluted in HBSS (0.1% w/V) (17 101 015, ThermoFisher) was conducted at 37 °C with agitation for five min. Any remaining undigested tissue was subjected to the same treatment until complete digestion was achieved. The isolated cells were then carefully filtered through a 40 µm filter to minimize cellular stress. The resulting filtrate underwent centrifugation twice at 150 g for ten min at room temperature (RT) and was resuspended in complete DMEM medium. The cells were pre‐plated in an incubator for two h on uncoated culture dishes to eliminate non‐CM cells, after which the CMs were plated in dishes at a density of 150 000 cells cm^−^^2^ using complete DMEM medium supplemented with Cytosine β‐D‐arabinofuranoside (10 µM, AraC; 147‐94‐4, ThermoFisher) to inhibit cell proliferation. After 24 h, the media was changed with complete DMEM medium and then every day for 5 days with DMEM medium supplemented with 1% P/S, 1% Amphotericin B, 2% Horse serum (16050‐130, ThermoFisher) and 0.5% HEPES Buffer Solution. After 18 h, the CMs reached confluence and began to beat spontaneously.

### Mouse Experiments

This research complies with all relevant ethical regulations. Experiments involving mouse xenografts received approval from the ethics committee at UCLouvain (approval ID 2020/UCL/MD032) and were conducted in accordance with national care regulations. Mice were housed with cycles of 12 h light/12 h dark, temperature (21–24 °C) and humidity control (40–60%). Rj:NMRI‐Foxn1nu/nu 5‐week female mice (Janvier) were accommodated during two weeks to the specific diets, isocaloric AMF butter‐containing control (Safe, U8978P0177) or PUFA‐rich anchovy/sardine oil‐containing (SAFE, U8978P0167). Control and shACOX1 SiHa cancer cells were washed twice with PBS, resuspended in 0.9% NaCl, and 2 × 10^6^ shACOX1 cells were injected in the left flank while control cells were injected in the right flank of each mouse. Similarly, 2 × 10^6^ FaDu cancer cells were injected into the right flank of each mouse. When tumors reached around 35–50 mm^3^, mice were treated with MPC inhibitor (7ACC2, 3 mg kg^−1^) diluted in DMSO by intraperitoneal injection for 5 days. shACOX1 SiHa xenograft mice were maintained under 7ACC2, while FaDu xenograft mice were cotreated with ACOX1 inhibitor (TDYA, 10 mg kg^−1^) for 5 days per week. Control groups were treated in parallel with equivalent vehicles. Mice were weighed, and tumors were measured by the same person 3 times per week using a calliper. Tumor volumes of mice did not exceed the limits authorized by the local ethical committee. The experiment ended when tumors reached ten times the initial volume, mice were sacrificed, and livers and tumors were collected for downstream processing (IHC/multiplex).

### Human Serum

Five healthy volunteers were recruited to participate in the clinical study Omegaserum (study 020/16DEC/622) performed at the “Centre d'investigation Clinique en nutrition” (CICN, Louvain‐la‐Neuve), which received the approval from the ethical committee of the Saint‐Luc University Hospital. Each volunteer received a box of MorDHA capsules (Minami) and was asked to take six capsules daily for one week (3840 mg ω3 PUFA). 100 mL of blood was drawn before and after ω3 supplementation. The blood was then centrifuged to collect the serum, which was used in organoid experiments. In parallel, the lipid analysis, lipid extraction from human serum, and data analysis methods were previously described.^[^ ^26^ ^]^ Briefly, gas chromatography‐flame ionization detection (GC‐FID) was used to quantify fatty acids as phospholipids, neutral lipids, and free fatty acids.

### Patient‐Derived CRC Organoids

The CRC organoid model was derived from colon cancer tissue obtained from a patient who underwent surgery at Cliniques Universitaires St Luc in Brussels (ethics committee approval n° ONCO‐2015‐02 updated on 13‐05‐2019 following the principles of the Declaration of Helsinki), as previously described.^[^ ^45^ ^]^ Before collecting the tumor, the patient provided signed informed consent, and all personal and clinical data were strictly kept confidential by the researchers. The CRC organoid cultures were plated in 50‐µL domes of Cultrex Basement Membrane Extract, Type 2 (#3532‐010‐02, Bio‐Techne) and maintained in Advanced DMEM/F12 (12 634 010, Gibco) supplemented with 1% Glutamax (35 050 061, Gibco), 1% HEPES (15 630 080, Gibco), 1% P/S (15140‐122, Gibco), 1% B27 without vitamin A (12 587 010, Gibco), 1% N2 supplement (17 502 048, Gibco), 50 ng/mL EGF (#78 006, STEMCELL Technologies), 10 mM nicotinamide (N0636, Sigma‐Aldrich) and 1.25 mm N‐acetyl cysteine (A8199, Sigma‐Aldrich), hereafter called PDO media. The organoids were usually split every 7–10 days, and the medium was refreshed every 2–3 days. When splitting the organoids, 500 µL of Gentle Cell Dissociation Reagent (STEMCELL Technologies) was added to each well. The Cultrex dome was scraped and collected using a P1000 pipette, pipetting up and down to break up the organoids. The sample was then incubated on ice for 5 min before being centrifuged for 5 min at 400 g. The resulting pellet was suspended in an appropriate volume of Cultrex and placed in 50‐µL domes in a 24‐well plate (#3524, Costar, Corning). The plate was incubated at 37 °C for 15 min before adding 500 µL of PDO medium.

### Lentiviral Packaging and Stable Cell Line Construction

HEK293T cells were purchased from ATCC and cultured in DMEM (Gibco) supplemented with 10% FBS, 25 mm Glucose, and 2 mm Glutamax without P/S.

For shRNA‐mediated downregulation of ACOX1, HEK293T cells were transfected by using the Calcium Phosphate transfection kit (#CAPHOS‐1KT, Merck) technique with 4 different plasmids: pLV[shRNA]‐U6 from VectorBuilder, shACOX1 #1, shACOX1 #2, shACOX1 #3, and Scramble control (Table S1, Supporting Information). Mediums containing lentiviral particles were filtered at 0.45 µm, diluted at 1:2 with fresh medium, and used to transduce SiHa cell lines for 48 h. Transduced SiHa cells were selected by the addition of puromycin for 1 week.

For pHluorin2, the HCT116 cell line was transduced as described above using the plasmid pLV[Exp]‐EF1A of pHluorin2 (VectorBuilder) (Table S1, Supporting Information). The transduced cell line was selected by adding hygromycin for 3 weeks.

For Hyper3‐PTS1, SiHa, HCT116, and FaDu cell lines were transduced as described above using the plasmid pLV[Exp]‐EF1A of HyPer3, where the PTS1 (TCCAAGCTC) sequence was added at the C‐terminal position (VectorBuilder) (Table S1, Supporting Information). Transduced cell lines were selected by adding blasticidin for 2 weeks.

### Fatty Acid Preparation

FA/BSA complex was prepared as described previously.^[^ ^46^ ^]^ Briefly, (Palmitic acid (PA, 10–1600, Larodan), Oleic acid (OA, 10–1801, Larodan), Docosahexaenoic acid (DHA, 10–2206, Larodan) and C22:1 FA (10‐2201, Larodan) were complexed with FA‐free BSA (A7030, Merck) in 150 mm NaCl solution to obtain a fatty acid/BSA ratio of 4:1 (mol:mol). Aliquoted fatty acids were stored at −80 °C, protected from light, and used directly upon thawing by adding the required concentrations to the wells.

### ^14^C Fatty Acid Uptake

Cells were plated in a 12‐well plate to reach 80–90% confluence after 24 h. ^14^C‐FA was mixed with free‐FA BSA in 7.4 serum‐free medium to obtain 1 µCi mL^−1^, and cold FA/BSA was also added to obtain a final concentration of 50 µm FA. Cells were maintained at 37 °C and washed with a 7.4 serum‐free medium. A ^14^C‐FA solution was added with a delay between wells to keep the same time exposure to ^14^C‐FA during the kinetics. Half‐plate was acidified by decreasing 0.2 pH units every 5 min during the kinetic by adding 1 m HCl, while water was added to the other half. Every 5 min, one well of each condition was washed with cold PBS (PBS‐1A, Capricorn) and stopped by adding 0.1 m NaOH lysis solution. Protein quantification was performed using the Bradford assay for normalization. After shaking, samples were mixed with MicroScint 20 (PerkinElmer) in OptiPlate‐96 for 2 h before reading with Topcount NXT Microplate scintillation and luminescence counter (PerkinElmer).

### 2D Cell Treatments

For the case of chemical control of pH, 10 000 cells/well were plated in a 96‐well plate with homemade DMEM 7.4 or 6.5. After 24 h, cells were treated with 50 µm DHA, C22:1, OA, or equivalent BSA concentration as vehicle in combination with metabolic drugs 15 µm DGAT1i (A922500, HY‐10038, MedChemExpress) or 60 µm ACOX1i (10,12‐Tricosadiynoic acid, HY‐135425, MedChemExpress) or their equivalent DMSO concentration as vehicle, with their usual‐adapted pH or by swapping to the opposite pH during 72 h for DGAT1i and 24 h for ACOX1i. To determine the effect of a mild concentration of ACOX1i, cells were treated with 30 µm ACOX1i (DMSO as vehicle) and 50 µm DHA (BSA as vehicle) with a swap of pHe previously described during 72 h. To determine the type of cell death associated with the combination of 50 µm DHA and 40 µm ACOX1i in acidosis, 30 µm Z‐VAD‐FMK (#4 026 865, Bachem) for apoptosis inhibitor, 30 µm ferrostatin‐1 (#S7243, Selleckchem) or 20 µm α‐tocopherol (#T3634, Sigma Aldrich) for ferroptosis inhibitors, were added at the same time during the treatment. For the positive control of ferroptosis, cells were treated with 5 µm RSL3 (HY‐100218A, MedChemExpress) and either coupled with or without 20 µm α‐tocopherol.

For drug‐control of the pH, 5 000 cells per well were plated in a 96‐well plate with DMEM supplemented with 10% FBS, 25 mm Glucose, 2 mm Glutamine, and 22 mm HCO_3_^−^ with the pH adjusted to 7.4. After 24 h, cells were pretreated with 10 µm 7ACC2 (HY‐D0713, MedChemExpress) or DMSO as vehicle. 72 h later, half volume was refreshed to maintain 10 µm 7ACC2 and treat cancer cells with a final concentration of 75 µm DHA (or BSA as vehicle) in combination with metabolic drugs (30 µm DGAT1i, 60 µm ACOX1i, or DMSO as vehicle) during 72 h. For ACOX1 downregulation by shRNA, 10 000 cells per well were plated in 96‐well with homemade medium 7.4. After 24 h, cells were treated with 75 µm DHA (or BSA as vehicle) in pH 7.4 or 6.5 for 72 h.

At the end of the treatment, cell viability was quantified by PrestoBlue reagent (A13262, Invitrogen) according to the manufacturer's instructions.

### 3D Spheroid Models

HCT116 and FaDu were used to form spheroids. They were cultured in commercial DMEM media supplemented with 10% heat‐inactivated FBS, 25 mm Glucose, 2 mm Glutamax, and 1% P/S. 1 000 cells per well were seeded in 96‐well ultra‐low attachment plates (7007, Costar) and maintained in culture for 7 days before the addition of 10 µm 7ACC2 as a pretreatment (or DMSO as vehicle). After 4 days, spheroids were treated with 100 µm DHA (or BSA as vehicle) and 60 µm ACOX1i (or DMSO as vehicle). To follow the cytotoxicity, Cytotox Green Reagent (#4633, Sartorius) was added to the medium, and the pictures were captured using Incucyte (#SX5, Sartorius) with a 10x objective. The fluorescence intensity was quantified using Incucyte Software.

### Label‐Free Live Cell LD Quantification

SiHa 7.4 > and 6.5 > were plated in the special 96‐well plate for NanoLive to reach 80–90% confluence the day after. Cells were maintained with their daily‐adapted pH or swapped with the opposite pH and treated with 25 µm DHA or an equivalent concentration of FA‐free BSA. Each condition was captured every 10 min for a total acquisition of 290 min by CX‐A (NanoLive). The ratio of the total LD area to the parent cell area was calculated by the software from the manufacturer (Smart lipid Droplet AssayLive, NanoLive), and the early velocity of LD synthesis was measured by non‐linear regression method with the software GraphPad Prism 9.

### Oil Red O staining

A stock solution of ORO was produced at 0.8 g.mL^−1^ in 100% isopropanol and kept for 6 months. At the end of the experiment, cells were washed with PBS and fixed with 4% PFA for 20 min at RT. Then, cells were washed, and 60% isopropanol solution was added for 5 min at RT. The staining solution was made by mixing the stock solution with ultrapure water 3:2 (vol: vol), filtered twice with a 0.22 µm filter, and added to the cells for 20 min at RT. Cells were washed once with 60% isopropanol solution and rinsed with tap water until the water was colorless. Then, cells were stained with hematoxylin for 5 min at RT, washed with water, and sealed with a mounting medium (Dako). The staining was visualized using AxioImager.z1‐ApoTome1 (Zeiss), objective 63X.

### LD Quantification

For ORO staining, 8‐well chambers (80 841, Ibidi) were coated with poly‐L‐lysine (A‐003‐M, Merck), and 20 000 cancer cells were plated and treated with PA, OA, or DHA (or equivalent FA‐free BSA as vehicle) in a homemade medium adjusted at the indicated pHe for 24 h at 37 °C, 5% CO_2_. After staining with ORO, the percentage of pixels associated with LD was quantified using ImageJ. The thresholding was calculated by AutoThreshold (MaxEntropy), the percentage area of LD was determined and normalised by the number of nuclei, as previously described.^[^ ^26^ ^]^ Similarly, LD quantification was performed in bovine aortic endothelial cells and cardiomyocytes cultured in either 21% or 1% O_2_ conditions (H35 Hypoxystation, Don Whitley Scientific).

For live cell BODIPY staining, 20 000 7.4 > cancer cells were seeded in µ‐slide 8 well high (80 806, Ibidi) and treated the day after with 7.4 or 6.5 pH medium containing 50 µm DHA in combination or not with 2 µg mL^−1^ JC63.1 (ab23680; Abcam), 10 µm SSO (SML2148, Merck), 2 µm FATP‐1‐IN (HY‐141699, MedChemExpress) or 2 µm Lipofermata (HY‐116788, MedChemExpress). After 6 h, cells were wash with warmed PBS twice and incubate with a solution of 2 µm BODIPY 493/503 in PBS during 20 min at 37 °C and 5% CO_2_. After washing with warmed PBS, cells were maintained in 7.4 homemade medium without phenol red, containing 1% FBS and 1 µg mL^−1^ Hoechst 33 342 (H3570, Invitrogen) at 37 °C, 5% CO_2_ during live cell microscopy acquisition. Pictures were taken with a LSM 800 confocal microscope, objective 40x, Ex = 488 nm / Em = 530 ± 30 nm for BODIPY and Ex = 405 nm / Em = 450±25 nm for Hoechst. The percentage of pixels associated with LD was quantified using ImageJ. The threshold was determined to discriminate the LDs from the background and applied to the different conditions of the same cell line and experiment. The percentage area of LD was normalised by the number of nuclei.

### pH and Hypoxia

150 000 cancer cells were plated in a 6‐well plate with DMEM supplemented with 10% FBS, 25 mm Glucose, 2 mm Glutamine, 1% P/S and 22 mm HCO_3_^−^ with the pH adjusted at 7.4 or 8.4 and maintained at 21% O_2_ or 1% O_2_ (H35 Hypoxystation, Don Whitley Scientific), 5% CO_2_ and 37 °C. After 72 h, half volume was refreshed to treat cells with a final concentration of 25 µm DHA during 24 h. After incubation, the pH of the media was quickly quantified using a pH meter. For the LD quantification, 20 000 cells were plated in 8‐well chamber slides and treated similarly. At the end of the treatment, the cells were stained with ORO. The percentage of pixels associated with LD was quantified using ImageJ.

### pH Control by Metabolic Drugs

150 000 cancer cells were plated in a 6‐well plate with DMEM supplemented with 10% FBS, 25 mm Glucose, 2 mm Glutamine, 1% P/S, and 22 mm HCO_3_^−^with the pH adjusted to 7.4. After 24 h, the cells were treated with 10 µm 7ACC2, 15 mm DCA (HY‐Y0445A, MedChemExpress), or equivalent DMSO as vehicle for 72 h. Half the volume was refreshed to treat cells with a final concentration of 25 µm DHA (or BSA as vehicle) while maintaining 7ACC2 and DCA concentrations. After 24 h, the pH of the media was quickly quantified using a pH meter. For the LD quantification, 20 000 cells were plated in 8‐well chamber slides (80 841, Ibidi) and treated similarly. At the end of the treatment, the cells were stained with ORO. The percentage of pixels associated with LD was quantified using ImageJ and normalized with the control condition.

### Flow Cytometry

60% confluence of 6‐well plate of 6.5 pH‐adapted HCT116 and SiHa cells were treated with 50 µm DHA and/or 60 µm ACOX1i, DMSO, and FA‐free BSA were added as vehicles. For cell death measurements, cells were collected after 8 h of treatment and stained with 7‐AAD (559 925, BD Biosciences, 1:1000) and FITC Annexin V (51‐65874X, BD Biosciences, 1:20) for 15 min at RT according to the manufacturer's recommendation. Cells were acquired by flow cytometry using a FACSCanto II (BD Biosciences) with a gating strategy that excluded debris and double cells. For ROS quantification, cells were collected after 5 h of treatment and stained with 1.25 µm MitoSox Green (M36006, Invitrogen) or 1.5 µm CM‐H2DCFDA (C6827, Invitrogen) to quantify O_2_^•−^ and ROS, respectively, for 30 min at 37 °C and 5% CO_2_. For positive control of MitoSox, cells were incubated 24 h in hypoxia and stained under reoxygenation. Cells were acquired as described previously with an equivalent FITC channel.

### Immunofluorescence Staining

To validate the peroxisomal localization of HyPer3, HCT116 / 6.5 cells transduced with HyPer3‐PTS1 were washed twice with PBS and fixed with 4% PFA for 20 min at RT. After washing, cells were permeabilized with 0.1% Triton‐X100 for 20 min at RT, followed by three washes. Then, nonspecific sites were blocked by adding a 2% BSA solution for 1 h at RT before incubation with an ABCD3 (PMP70) antibody (SAB4200181, Merck, 1:1000) diluted in 0.1% BSA overnight at 4 °C. After incubation, the cells were washed 3 times with PBS and then incubated with an AlexaFluor 647‐conjugated anti‐mouse secondary antibody (A21236, Invitrogen, 1:600) and DAPI (D9542, Merck) diluted in 0.1% BSA for 1 h at RT. Finally, cells were washed with PBS before being imaged using a LSM 800 confocal microscope, with AiryScan mode, objective 63x, Ex = 640 nm / Em = 670 ± 25 nm for ABCD3, Ex = 488 nm / Em = 535±25 nm for Hyper3 and Ex = 405 nm / Em = 450±25 nm for DAPI.

To perform the immunostaining of CHOP, 60% confluence of µ‐slide 8‐well high (80 806, Ibidi) of acidic‐adapted cancer cells were treated with 50 µm DHA and 60 µm ACOX1i (or equivalent BSA and DMSO as vehicles) for 4 h. Before a high rate of cell death, cells were washed, fixed, and stained as previously described using a CHOP antibody (L63F7, Cell Signaling, 1:600) and an AlexaFluor 488‐conjugated anti‐mouse secondary antibody (A32766, Invitrogen, 1:500), along with DAPI. After mounting with Dako Fluorescent mounting media (S3023, Agilent), images were captured using a LSM 800 confocal microscope with a 40x objective. The excitation wavelengths were 488 nm for AlexaFluor 488 and 405 nm for DAPI, with emission wavelengths of 530 ± 30 and 450 ± 25 nm, respectively. The mean fluorescent intensity of nuclear CHOP was calculated using ImageJ. A Nuclei Mask was drawn manually with DAPI picture and applied to quantify the mean fluorescent intensity of CHOP for each nucleus.

### Macropinocytosis

When cancer cells 7.4 > and 6.5 > reached around 60% confluence, they were maintained in homemade serum‐free DMEM 7.4 and 6.5 with 1 mg.mL^−1^ TMR‐Dextran 70 kDa (D1818, Invitrogen) for 30 min at 37 °C. After incubation, cells were washed four times with cold PBS and fixed with 4% formaldehyde. Nuclei were stained by incubation with DAPI. Images were captured in PBS using an LSM 800 confocal microscope with a 40x objective. The excitation wavelengths were 561 nm for TMR‐Dextran and 405 nm for DAPI, with emission wavelengths of 590 ± 25 and 450 ± 25 nm, respectively. After determining a threshold for all conditions, the pixel percentage of Dextran per cell was calculated using ImageJ and normalized to the cell line maintained in 7.4 medium.

### Intracellular pH Measurements

Ringer solutions at pH 7.4 and 6.5 were prepared as previously described.^[^ ^24^ ^]^ Before starting the kinetic experiment, pH verification was done after adding 50 µm uncomplexed DHA (solubilized in EtOH) to validate the pH stability of the Ringer solutions. pHluorin2 cells were plated in µ‐slide 8‐well high (80 806, Ibidi) to reach 80% confluence two days later. For the acidification kinetics, cells were washed once with heated PBS before adding heated Ringer solution at pH 7.4 or 6.5 and supplemented or not with 50 µm uncomplexed DHA (EtOH as vehicle). For the acidification endpoint, cells were maintained in 7.4 or 6.5 phenol‐free homemade medium and treated with 50 µm DHA and 15 µm DGAT1i (BSA and DMSO as vehicles) for 24 h before taking pictures. pHluorin2 live cells were directly imaged with a Zeiss LSM800 confocal microscope using a 40X objective at 37 °C and 5% CO2, during 2h40 for the kinetic. Emission was fixed at 509 ± 25 nm, and pictures were acquired with excitation at 405 and 488 nm. Upon acidification, emission at 488 nm excitation increases, while emission at 405 nm excitation decreases. Analysis of the pictures and calculation of the Ex_488_/Ex_405_ ratio were performed using ZEN Blue software from Zeiss.

### Membrane Fluidity Measurements

20 000 SiHa cells were seeded in µ‐slide 8‐well high (80 806, Ibidi) and treated the day after with 50 µm DHA in combination or not with 30 µm DGAT1i or 30 µm ACOX1i in 7.4 and 6.5 phenol‐free homemade medium. After 24 h, 2 µm Laurdan (HY‐D0080, MedChemExpress) was added to the medium overnight. For ACOX1i, the cells were stained with 1 µm ER‐tracker Red (E34250, Invitrogen) for 20 min at 37 °C, 5% CO_2_ before the microscopy session. After washing, homemade medium without phenol red was added and live cells were imaged with a Zeiss LSM800 confocal microscope with a 40X objective at 37 °C and 5% CO_2_. Excitation was fixed at 405 nm, and two emissions at 437.5 ± 37.5 and 524.5 ± 25.5 nm were captured, representing the rigid and fluid membrane states, respectively. For ER‐tracker red, Ex = 561 nm and Em = 615 ± 25 nm. The plasma membrane and Endomembrane were manually selected to avoid LD influence, and fluorescence intensity for both emissions was analyzed for each cell using ImageJ. Similarly, for ACOX1i experience, ER‐tracker staining was used to define a Mask corresponding to the pixels of ER membrane. Via RStudio software, Generalized Polarization (GP) was calculated as previously described^[^ ^31^ ^]^ to represent the membrane fluidity state.

### Peroxisomal H_2_O_2_ Measurements

20 000 HyPer3‐PTS1 cells were seeded in µ‐slide 8‐well high (80 806, Ibidi). The day after, cells were treated with 50 µm DHA or C22:1, either in combination or not, with 15 µm DGAT1i or 60 µM ACOX1i in 7.4 and 6.5 phenol‐free homemade medium for 24 h for DGAT1i and 6 h for ACOX1i. Then, live cells were imaged with a Zeiss LSM800 confocal microscope using a 40X objective at 37 °C and 5% CO_2_. Emission was fixed at 535 ± 25 nm, and pictures were acquired with excitation at 405 and 488 nm, respectively, corresponding to the reduced and oxidized forms. With ImageJ software, a threshold was applied to the oxidized form picture to discriminate the signal from the background and apply it to all conditions. Then, a mask corresponding to the peroxisomal Hyper3 signal was determined and used to calculate the median of the Ex_488_/Ex_405_ ratio for each picture using RStudio.

### Endoplasmic Reticulum Morphology

Acidic‐adapted cancer cells were treated with 50 µm DHA, either in combination or not, with 60 µm ACOX1i (BSA and DMSO as vehicles, respectively) in 6.5 phenol‐free homemade medium for 5 h. Cells were washed with PBS and stained with 1 µm ER‐Tracker Green (E34251, Invitrogen) and 10 µg mL^−1^ Hoechst 33 342 (H3570, Invitrogen) in HBSS with Ca^2+^/Mg^2+^ (HBSS‐1A, Capricorn) for 30 min at 37 °C and 5% CO_2_. After washing, 6.5 phenol‐free medium was added, and live cells were imaged using a confocal microscope with a 40X objective at 37 °C and 5% CO_2_. The excitation wavelengths were 488 nm for ER‐Tracker green and 405 nm for Hoechst 33 342, with emission wavelengths of 530 ± 30 and 450 ± 25 nm, respectively.

### RNA Extraction and RT‐qPCR

Cancer cells were lysed with TRI Reagent solution (#TR118, Molecular Research Center) after DHA/ACOX1i treatment (using BSA/DMSO as vehicles) and prior to a high rate of cell death. Briefly, total RNA was recovered after chloroform separation (aqueous part), precipitation with isopropanol, washing with 70% ethanol, and resuspension in RNAse‐free water. The RNA quantity and quality were evaluated with a Nanodrop 1000 spectrophotometer (Thermo Fischer Scientific). 1 µg of total RNA was used for reverse transcription with RevertAid Reverse‐Transcriptase, oligo‐dT, and random hexamers (Thermo Fisher Scientific). Then, quantitative PCR amplification was performed using the CFX Connect Real‐Time PCR detection system (#1 855 201, Bio‐Rad), with GoTaq qPCR Master Mix (A6001; Promega) and sequence‐specific primers for the ACOX1, ATF3, CHAC1, DDIT3, HERPUD1, and HSPA5 genes (Table S2, Supporting Information). Relative gene expression was calculated using the ddCq method, with RLPL0 as the housekeeping gene.

### Western Blotting

Cancer cells were washed and lysed with RIPA buffer supplemented with a protease inhibitor cocktail (P8340, Merck) and phosphatase inhibitors (4 906 837 001, Merck), after treatment with DHA/ACOX1i (BSA/DMSO as vehicles) and before a high rate of cell death. Protein concentration was determined using the Pierce BCA protein assay (Thermo Fisher Scientific). Protein samples were mixed with Laemmli solution and heated at 95 °C for 5 min. 30 µg of protein were loaded, separated by SDS‐PAGE, and transferred onto PVDF membranes. Then, membranes were blocked with 5% skimmed milk diluted in TBS‐0.1% Tween 20 and immunoblotted overnight at 4 °C with specific primary antibodies, P‐p38 (#4511, Cell Signaling, 1:1000), p38 (#9212, Cell Signaling, 1:1000), P‐eIF2α (#9721, Cell Signaling, 1:1000), eIF2α (#2103S, Cell Signaling, 1:1000), P‐IRE1α (NB100‐2323, Novus Biological, 1:1000), IRE1α (#3294, Cell Signaling, 1:1000), Ubiquitin (#3936, Cell Signaling, 1:1000) and β‐actin (A5441, Merck, 1:10 000). After several washes with TTBS, membranes were incubated for 1 h at RT with horseradish peroxidase (HRP)‐conjugated secondary antibodies (Jackson Immunoresearch), and chemiluminescent signals were revealed by using ECL Western Blotting Detection Kit (GE Healthcare) on X‐ray films in a dark chamber. Semi‐quantitative analysis was done using ImageJ. For each protein, a selection square was drawn to contain the biggest band, and it was used to quantify the grey intensity of each band and the background. The measured intensities were inverted with the maximum intensity (255), and the background was subtracted. The ratio of the Phosphorylated form to the unphosphorylated form was calculated.

### Tumor Multiplex Immunolabeling and Liver Histochemistry

For histologic and immunolabeling analyses, the tumors and livers from FaDu xenografts in vivo experiment were collected and fixed in 4% PFA before inclusion in paraffin for sectioning for hematoxylin and eosin (H&E) staining, or immunolabeling, 5‐µm‐thick sections were used. Briefly, after deparaffinization and blocking of endogenous peroxidases with a 3% hydrogen peroxide solution, tissue sections were subjected to antigen retrieval using 10 mM citrate buffer, pH 6.0. The antigen retrieval was performed in a microwave following 4:30 min at 900 W, 15 min at 90 W, and 1:30 min at 900 W. After this, the samples were cooled down at RT before adding the blocking solution (BSA 5% in TBS‐Tween 1%) for 30 min at RT. After blocking, sections were incubated with different primary antibodies (anti‐vimentin (#5741S, rabbit, Cell Signaling, 1:400) and anti‐pan Cytokeratin (M3515, Mouse, Dako, 1:50)) for 1 h at RT. The secondary anti‐rabbit‐ or anti‐mouse‐HRP antibody (Dako, references K4003 and K43001, respectively) was applied to the sections for 40 min at RT. Staining was visualized by the addition of TSA and incubated for 10 min at RT. AF594 tyramine (B40957, Invitrogen, 1:200) was used for Vimentin, and CF430 Tyramide (#96 053, Biotium, 1:1000) was used for pan Cytokeratin. The staining for each antibody was done sequentially. Finally, the sections were counterstained with Hoechst 33 342 (H3570, Invitrogen, 1:1000), mounted in Dako Fluorescent mounting medium (S3023, Agilent), and scanned using an AxioScan slide scanner (Zeiss) at 20× magnification. The lasers used were 405 nm for Hoechst, 430 , and 590 nm for exciting the TSA fluorophores. Picture analysis was performed with QuPath software.

For liver section H&E, after deparaffinization and dehydration, the sections were stained with Hematoxylin and Eosin (Dako), then scanned with a Pannoramic Scan II (3D Histech) at 20X. The vacuole area was quantified after excluding blood vessels with QuPath software.

### Organoid Treatment

For the experiments, the organoids were processed into single cells using TrypLE Express (12 604 013, Gibco) and seeded with a density of 2500 cells/5 µL Cultrex matrix per well in a 96‐well plate in a PDO medium. After 3–5 days, organoids acquired their typical 3D structure and were further treated with either DMSO or 7ACC2 10 µm for 4 days using media containing 50% DMEM Glutamax (Gibco) and 50% AdvDMEM/F12 supplemented with 1% FBS. After this time, the organoids were treated with either vehicle (DMSO/BSA), ACOX1i (+BSA), DHA (+DMSO), or ACOX1i/DHA for an additional 4 days, either in media containing DMSO or 7ACC2. For the human serum experiments, organoids were cultured for 4 days in 50% DMEM Glutamax and 50% AdvDMEM/F12 supplemented with 10% patient serum before the consumption of omega‐3 capsules (serum before ω3, see Table 1 for details of healthy donors), treated with DMSO or 7ACC2 10 µm. After 4 days, the media was changed to Media + 10% serum before ω3 or Media + 10% serum after ω3 (from the same patient, but after 1 week of ω3 supplementation) and treated with vehicle (DMSO) or ACOX1i in DMSO or 7ACC2 10 µm‐containing media. The experiments where the organoids were treated in 7.4 or 6.5 media maintained the same treatment plan and concentrations by changing to the respective media instead of adding 7ACC2. The media were prepared for these experiments as described for the homemade 2D media. Then, to obtain a final pH of 6.5 or 7.4, the homemade media was mixed with 50% DMEM Glutamax and 50% AdvDMEM/F12 supplemented with 10% human serum.

Cell toxicity was assessed for all experiments using the CellTox Green Cytotoxicity Assay (G8742; Promega) by measuring fluorescence levels at the Spectramax device (Molecular Devices). Pictures of the fluorescence of the CRC#2 organoids treated with the compounds were obtained using a Zeiss Axiovert S100. Image analysis was performed using ImageJ (version 1.8.0).

For the pH quantification after pre‐acidification with 7ACC2, organoids were cultured with either DMSO or 7ACC2 10 µm for 8 days using media containing 50% DMEM Glutamax and 50% AdvDMEM/F12 supplemented with 1% FBS and 1% P/S. The media was refreshed after 4 days. After incubation, the pH of the media was quickly quantified using a pH meter.

### Lipidomics Analysis by Mass Spectrometry

Formic acid, LC‐MS grade ammonium formate, acetonitrile, water, and isopropanol of Ultra LC‐MS grade were supplied by Fisher Scientific (Brussels, Belgium). SPLASH Lipidomix II deuterated internal standard mix was obtained from Avanti Polar Lipids (Alabaster, AL, USA).

SiHa cells (at pH 7.4) were plated at 20% confluence in a 10 cm Dish. 24 h later, the medium was changed to 7.4 or 6.5 home‐made mediums with 50 µM of the corresponding fatty acid, 7.5 µm DGAT1i or 15 µM ACOX1i (DMSO as vehicle). The experience with 50 µm Methyl‐DHA (20‐2206, Larodan) was compared with cells supplemented with DHA solubilized in EtOH (and not coupled with BSA). After 24 h of incubation, cells were washed with cold PBS, and after removing excess PBS, cells were directly frozen with the contact of the dish bottom to liquid nitrogen. Frozen cells were harvested by scraping, and biomass was weighed for normalization.

Extraction was performed via a monophasic MeOH/IPA/MTBE (1/1/1.33 v/v/v) extraction protocol. Briefly, 1 mL of ice‐cold extraction solvent, 20 µL of internal standard mix, and 20 µL of BHT (20 µg mL^−1^) were added to the samples, and they were ultrasonicated for 10 min. The samples were then spun down for 10 min (20 000 g at 4 °C), the supernatant removed and evaporated to dryness with a high‐performance evaporator (Genevac EZ‐2) (Genevac, Ipswich, UK) under nitrogen. Reconstitution was performed in 30 µL MeOH. After reconstituting, the sample was briefly vortexed for 10 s and spun down for 10 min (20 000 g at 4 °C). The supernatant was taken and transferred to UHPLC vials for analysis, equipped with silanized micro‐inserts for maximum recovery.

µLC‐MS analysis was performed on a ThermoFisher (Dilbeek, Belgium) Vanquish Horizon system equipped with a binary pump, autosampler, and thermostated column compartment coupled to an Orbitrap Exploris 240 mass spectrometer. Chromatographic separation was performed on a Waters (Antwerp, Belgium) Acquity CSH C18 column (100×1.0 mm, 1.7 µm). Mobile phase A was composed of 60/40 (H_2_O/ACN, v/v), containing 0.1% LC‐MS grade formic acid and 10 mm LC‐MS grade NH_4_FA. Mobile phase B was composed of 1/19/80 (H_2_O/ACN/IPA v/v/v) containing 0.1% LC‐MS grade formic acid and 10 mm of LC‐MS grade NH_4_FA. The chromatographic gradient was as follows: (0.0 min 30% B, 1.0 min 30% B, 1.01 min 57.5% B, 10.0 min 57.5% B, 25.0 min 68% B, 30.0 min 99% B, 40.0 min 99% B, 40.01 30% B, 45.0 min 30% B) and a flow rate of 50 µL min^−1^ was used. A constant column temperature of 40 °C was maintained throughout the entire analytical run, and the injection volume was 2 µL. The autosampler was kept at 4 °C. Ion source parameters were as follows: Sheathe gas (25, nitrogen), aux gas (5, nitrogen), sweep gas (0 psi, nitrogen), ion transfer tube temperature (320 °C), ion source voltage +3500 V and ‐2500 in negative mode. Analytes within a range of 100–1500 m/z were recorded with a mass resolving power of 11 250 and the RF lens setting was at 70%. An isolation window of 2 m/z was used. Microscans were set to 1, AGC‐Target value was set to 1e6 with a Maximum Injection Time of 100 ms. During MS/MS, analytes were recorded at a resolution of 11 250 with collision Energy normalized to 25/30/35%. Microscans were set to 1, AGC‐Target value was set to 1e5 with a Maximum Injection Time of 50 ms. Data processing, comprising peak integration and regression, was performed with MS‐DIAL v. 4.9.221218^[^ ^47^ ^]^ and FreeStyle for further manual inspection. Lipidomics data processing in MS‐DIAL included MS/MS feature identification and peak alignment across the sample sequence. A retention time deviation window of 1 minute was applied for alignment. Feature identification was performed against an in‐house lipidomics spectral library using a similarity score cutoff of 80%, based on six parameters: retention time, m/z, isotopic pattern, and three MS/MS‐based scores (dot product, reverse dot product, and fragment presence). Peak intensity threshold was set to 1000 cps, with a mass tolerance of 0.01 Da for MS1 and 0.05 Da for MS/MS. To further minimize misannotations, expected intra‐class elution behaviour in reversed‐phase chromatography (based on acyl chain length and degree of unsaturation) was also considered. Normalization was performed using the B‐MIS approach in RStudio (R Foundation for Statistical Computing, Vienna, Austria), based on deuterated internal standards from the SPLASH Lipidomix II mixture.

### Glycerol Quantification

Cancer cells were plated at 20% confluence in a 10 cm dish. The day after they were treated with 10 µm 7ACC2, 15 mm DCA, or an equivalent amount of DMSO as a control. After 72 h, the cells were washed with cold PBS, and after removing excess PBS, they were directly frozen with the dish bottom to contact with liquid nitrogen. Frozen cells were harvested by scraping, and biomass was weighed for normalization. Extraction was performed with 1 mL of H_2_O:MetOH (V:V, 50:50) during 15 min. Then, the samples were ultrasonicated for 10 min and centrifuged for 10 min (20 000 g at 4 °C). The supernatants were kept and evaporated to dryness with a high‐performance evaporator (Genevac EZ‐2) (Genevac, Ipswich, UK) under nitrogen. The samples were resuspended in 50 µL of PBS, then glycerol and 3‐P‐glycerol were quantified using a microdialysis analyzer (ISCUS Flex, µDialysis) with the glycerol reagent kit (P000025, µDialysis).

### Statistical Analysis

Statistical analyses were performed using GraphPad Prism 10 by using unpaired Student's t‐test, one‐way ANOVA with Tukey's multiple comparison test, and two‐way ANOVA with Tukey's multiple comparison test. Statistical significance is indicated as follows: exact *p*‐values are provided when p‐values are between 0.001 and 0.5, while ns and *** represent, respectively, p‐values > 0.5 and *p*‐values ≤ 0.001. Aberrant outlier values were eliminated after confirmation using a mathematical outlier tool with the ROUT method of regression (GraphPad) and a coefficient of Q = 1%. No statistical method was used to predetermine the sample size. Mice were randomly assigned to each group. The investigators were not blinded to allocation during experiments and outcome assessment.

## Conflict Of Interest

The authors declare no conflict of interest.

## Author Contributions

S.I. and M.V.G. have contributed equally to this work. O.F. and C.D. have equally supervised this work. S.I., M.V.G., C.D., and O.F. performed conceptualization; S.I., M.V.G., S.A.S., L.A., D.M., H.V., E.H., C.B., D.B., C.A.A., and B.P. performed methodology; S.I., M.V.G., S.A.S., F.L., E.D., and C.G. performed investigation; C.D. and O.F. performed funding acquisition; C.D. and O.F. performed supervision; S.I., M.V.G., C.D., and O.F. wrote original draft.

## Supporting information



Supporting Information

## Data Availability

The data that support the findings of this study are available in the supplementary material of this article.
