# Supplementary material for: Acidosis Forces Fatty Acid Uptake and Metabolism in Cancer Cells Regardless of Genotype
Source: Adv Sci (Weinh). 2025 Jul 29;12(40):e05436. doi: 10.1002/advs.202505436 (PMC12561199; doi:10.1002/advs.202505436)
Supplement: Supplementary file 1 — Supporting Information [file ADVS-12-e05436-s001.pdf]

Supporting Information

Acidosis forces fatty acid uptake and metabolism in cancer cells regardless of genotype

Sébastien Ibanez[1,§,*], Maria Virginia Giolito[1,§], Sultan Al-Siyabi[1], Kristian Serafimov[1,2], Florine Laloux[1], Emeline Dierge[1,3], Léo Aubert[1], Céline Guilbaud[1], Selena Kaye[1], Paula Varon[1], Dorothée Marchand[1], Hanne Vlieghe[4], Emmanuel Hermans[5], Caroline Bouzin[6], Davide Brusa[7], Christiani Andrade Amorim[4], Barabara Pachikian[8], Yvan Larondelle[3], Cyril Corbet[1,8], Chantal Dessy[1,#] and Olivier Feron[1,2,8, #,*]

Suppl. Figure 1.

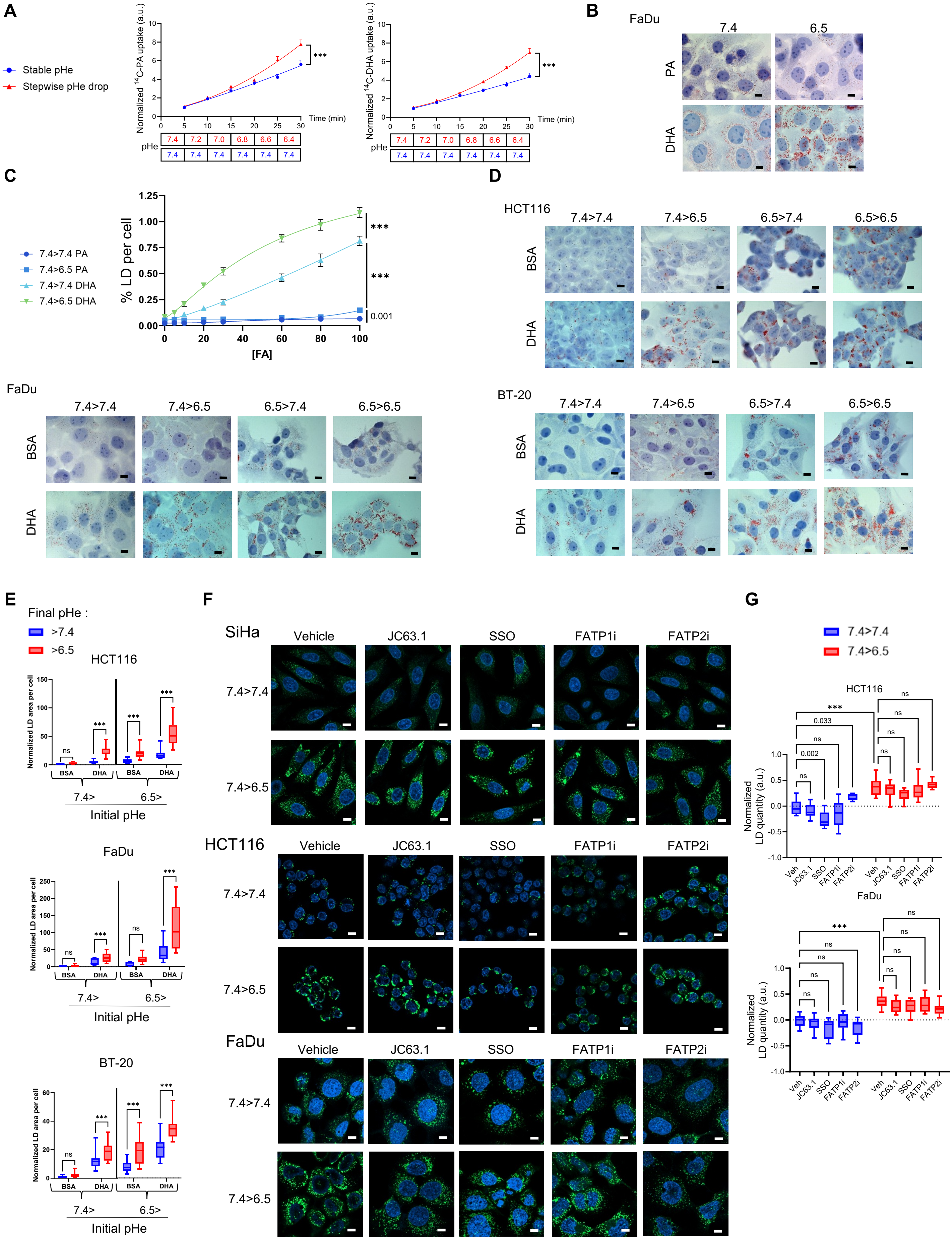

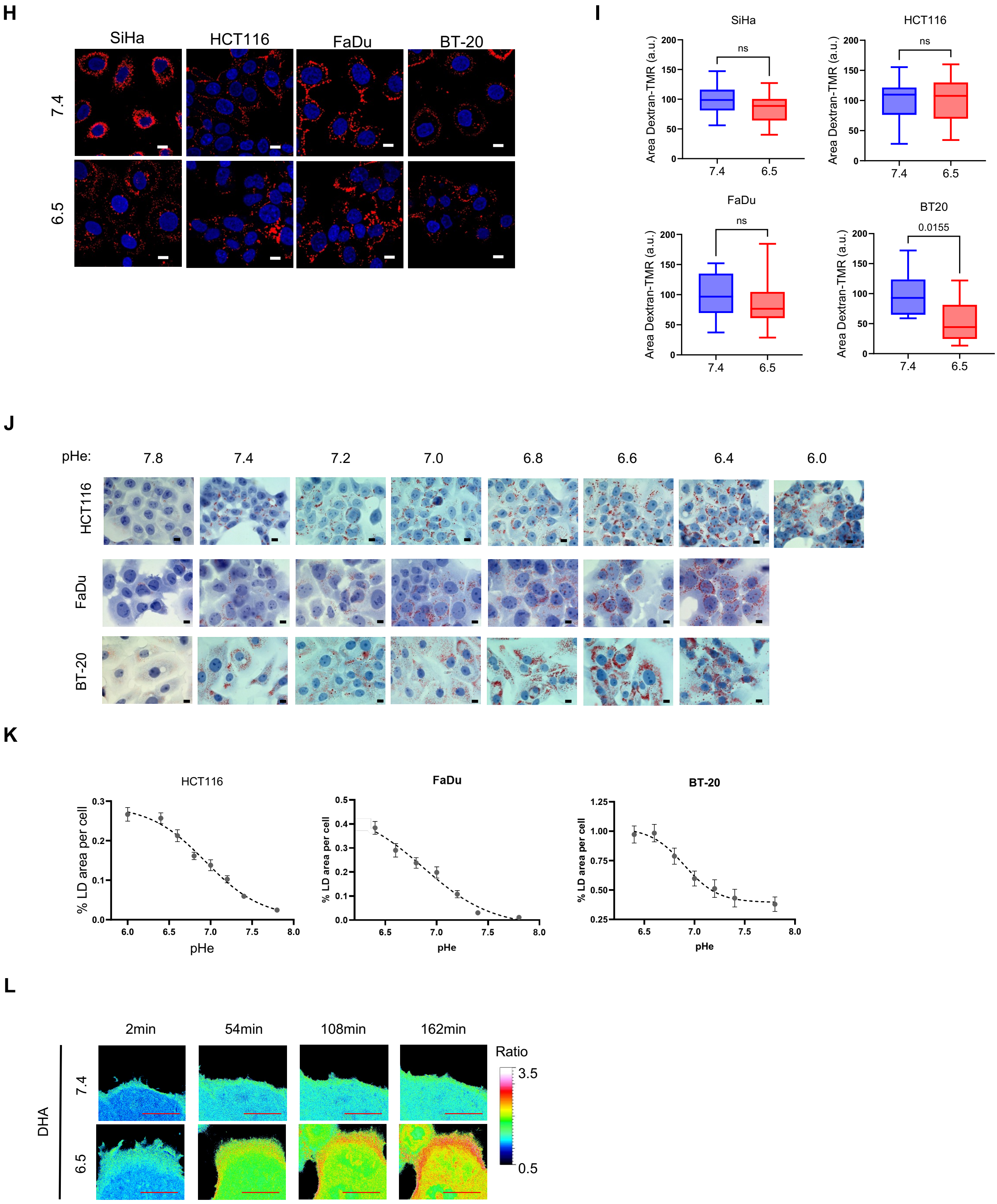
H
SiHa
HCT116
FaDu
BT-20
7.4
6.5
I
SiHa
HCT116
FaDu
BT20
Area Dextran-TMR (a.u.)
ns
0.0155
0
50
100
150
200
7.4
6.5
J
pHe:
7.8
7.4
7.2
7.0
6.8
6.6
6.4
6.0
HCT116
FaDu
BT-20
K
HCT116
FaDu
BT-20
% LD area per cell
pHe
0.0
0.1
0.2
0.3
0.4
0.5
0.25
0.50
0.75
1.00
1.25
6.0
6.5
7.0
7.5
8.0
L
2min
54min
108min
162min
Ratio
3.5
0.5
DHA
7.4
6.5

Figure S1. Extracellular pHe determines the extent of FA uptake. A. Normalised quantification of ^{14}C fatty acid uptake during stepwise medium acidification in FaDu cells (vs. maintaining stable pHe) for palmitic acid (PA) (left graph) and docosahexaenoic acid (DHA) (right graph) (N=3, n = 1). **B-C.** Representative pictures (B) and quantification (C) of ORO-stained lipid droplets (LD) in FaDu cells maintained at pHe 7.4 (7.4>7.4) or shifted to pHe 6.5 (7.4>6.5) upon supplementation for 24 hours with the indicated concentration ranges of PA or DHA (N=3, n=5 fields). **D-E.** Representative end-point pictures (D) and quantification (E) of LD in HCT116, FaDu, and BT-20 cells maintained at the indicated pHe (7.4>7.4 and 6.5>6.5) or following pHe swapping (7.4>6.5 and 6.5>7.4) and supplemented with 50 µM DHA or FA-free BSA as vehicle (N=2, n=10 fields). **F-G.** Representative pictures (F) and quantification (G) of BODIPY-stained LD in SiHa, HCT116, and FaDu cells maintained in 7.4 (7.4>7.4) or shifted in 6.5 (7.4>6.5) upon supplementation for 6 hours with DHA and coupled with FA transporter inhibitors (2 µg/mL JC63.1, 10 µM SSO, 2 µM FATP1i, 2 µM FATP2i) or DMSO as vehicle (N=3, n=5). **H-I.** Representative pictures (H) and quantification (I) of macropinocytosis in physiological and acidic adapted SiHa, HCT116, FaDu, and BT-20 cell lines stained with 1 mg.mL^{-1} TMR-Dextran (70kDa), data normalised to 7.4 condition (N=2, n=5 fields). **J-K.** Representative pictures (J) and quantification (K) of ORO-stained LD in HCT116, FaDu, and BT-20 cells exposed to the indicated pHe and supplemented with 50 µM DHA or FA-free BSA as vehicle (N=2, n=5 fields). **L.** Representative pictures with a focus on the plasma membrane from pHluorin2 HCT116 exposed for 160 min to 50 µM DHA. All scale bars: 10 µm. Graphs are presented as mean ± SEM (A, C, K) or min to max whisker plots, range = Q1 to Q3, and line = median (E, G, I). N = biological replicates, and n = technical replicates. ns = non-significant; *** = $p<0.001$. Significance was determined by an unpaired t-test (I) and a two-way ANOVA with Tukey's multiple comparison test (A, C, E, G); p-values given for A and C are the column factor.

Suppl. Figure 2.

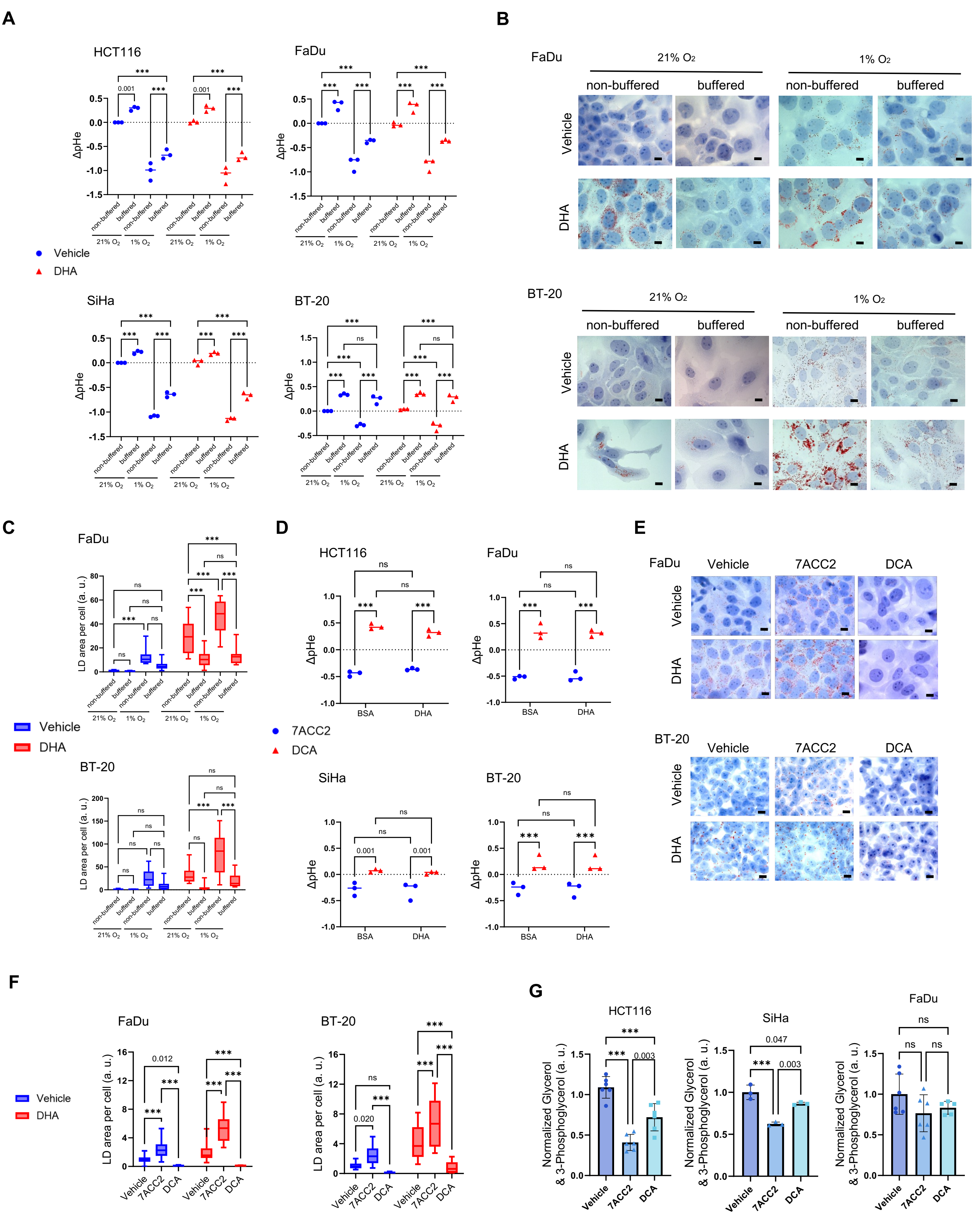

Figure S2. Extracellular acidification by hypoxia or stimulated glycolysis enhances DHA uptake across cancer cell types. A. External pH quantification of SiHa, HCT116, FaDu, and BT-20 cells maintained in normoxia or hypoxia (1% O_2) in either non-buffered or buffered bicarbonate buffer and then supplemented with 25 µM DHA or FA-free BSA vehicle (N=3, n = 1). **B-C.** Representative pictures (B) and quantification (C) of ORO-stained LD in FaDu and BT-20 cells supplemented with 25 µM DHA or FA-free BSA as vehicle in the indicated conditions. Data are normalized to control the non-buffered 21% O_2 condition (N = 2, n = 10 fields). **D.** External pH quantification normalised to the control of SiHa, HCT116, FaDu, and BT-20 cells supplemented with 25 µM DHA or FA-free BSA as vehicle after the indicated pharmacological treatments (DMSO as vehicle) (N=3, n = 1). **E-F.** Representative pictures (E) and quantification (F) of ORO-stained LD in FaDu and BT-20 cells supplemented with 25 µM DHA or FA-free BSA as vehicle after the indicated pharmacological treatments (DMSO as vehicle). Data are normalized to the control condition (N=2, n=10 fields). **G.** SiHa, HCT116, and FaDu cancer cells were treated for 72 h with 10 µM 7ACC2 or 15 mM DCA (DMSO as vehicle). The sum of 3-P-glycerol and glycerol was quantified by microdialysis assay after metabolite extraction (N = 6 for HCT116 and FaDu, N = 3 for SiHa). All scale bars: 10 µm. Graphs are presented as mean (A, D), min to max whisker plots, range = Q1 to Q3, and line = median (C, F) or as mean ± SD. N = biological replicates, and n = technical replicates. ns = non-significant; *** = $p<0.001$. Significance was determined by one-way ANOVA (G) and two-way ANOVA (A, C, D, F) with Tukey's multiple comparison test.

Suppl. Figure 3.

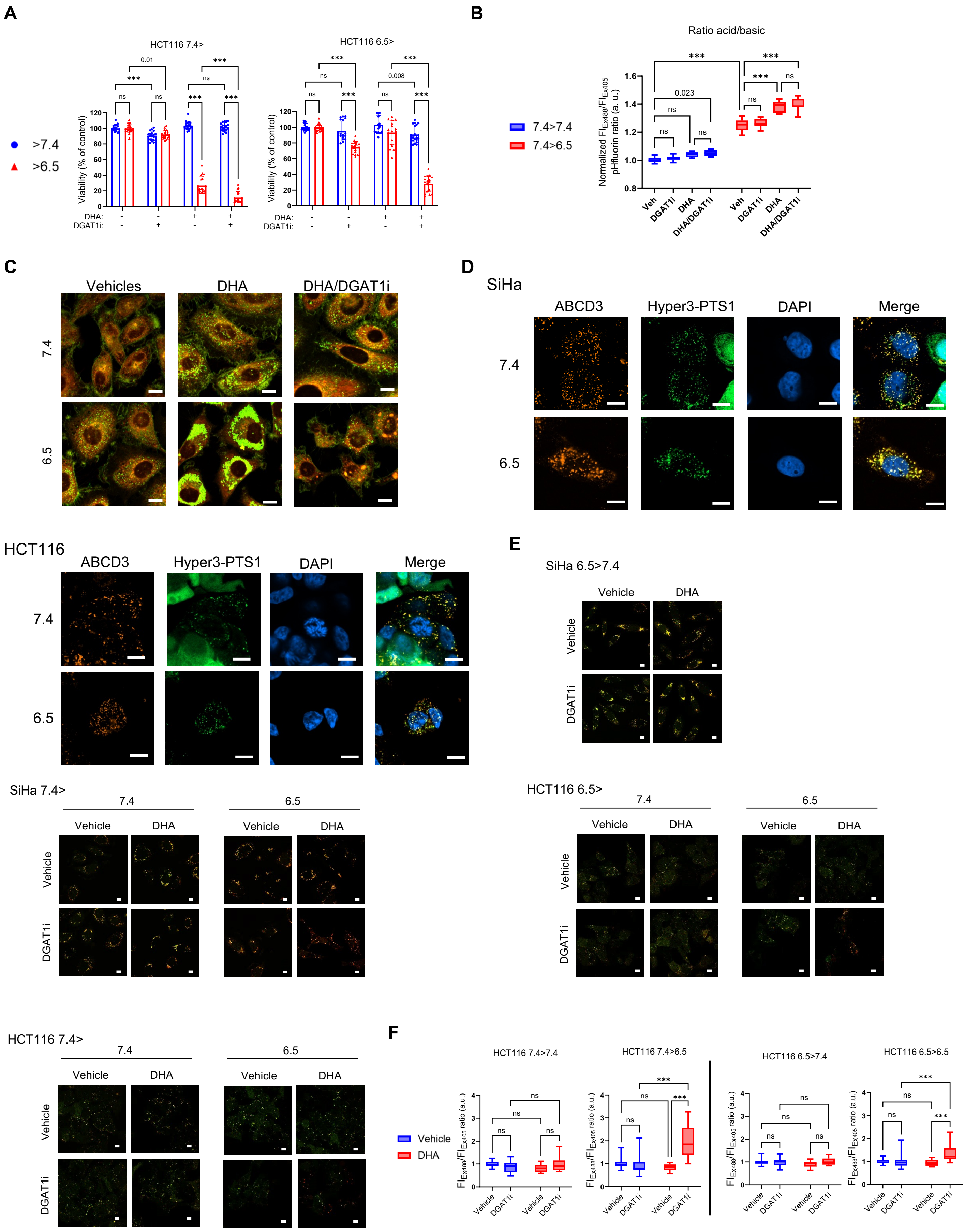

G

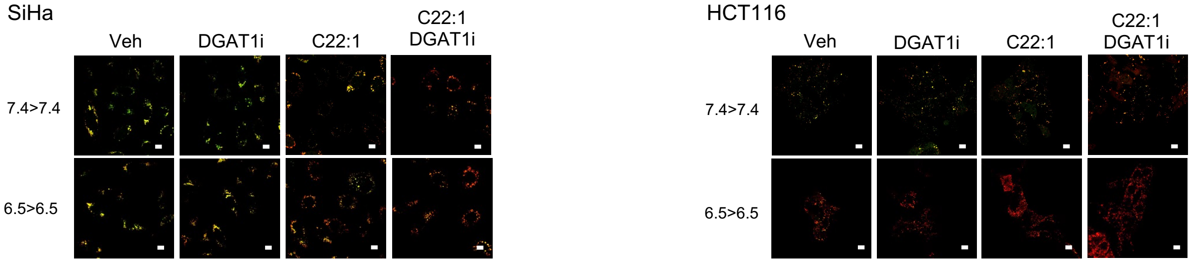
SiHa
Veh
DGAT1i
C22:1
C22:1
DGAT1i
7.4>7.4
6.5>6.5
HCT116
Veh
DGAT1i
C22:1
C22:1
DGAT1i
7.4>7.4
6.5>6.5

H

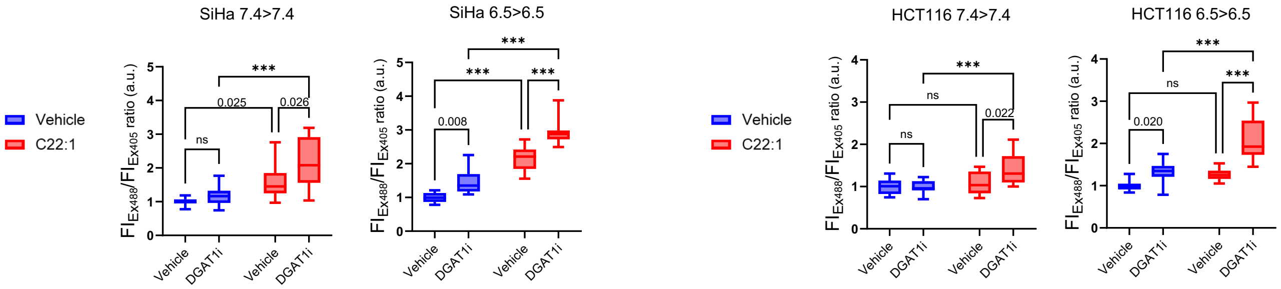
Vehicle
C22:1
SiHa 7.4>7.4
SiHa 6.5>6.5
HCT116 7.4>7.4
HCT116 6.5>6.5
FI$_{Ex488}$/FI$_{Ex405}$ ratio (a.u.)
ns
0.025
0.026
***
0.008
0.022
0.020
Vehicle
DGAT1i

I

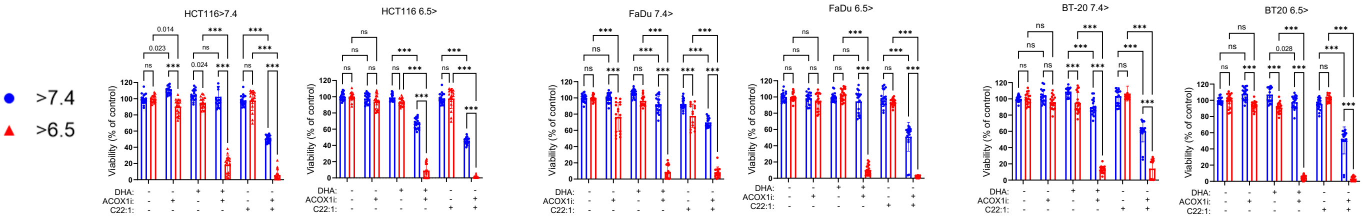
>7.4
>6.5
HCT116>7.4
HCT116 6.5>
FaDu 7.4>
FaDu 6.5>
BT-20 7.4>
BT20 6.5>
Viability (% of control)
DHA:
ACOX1i:
C22:1:

J

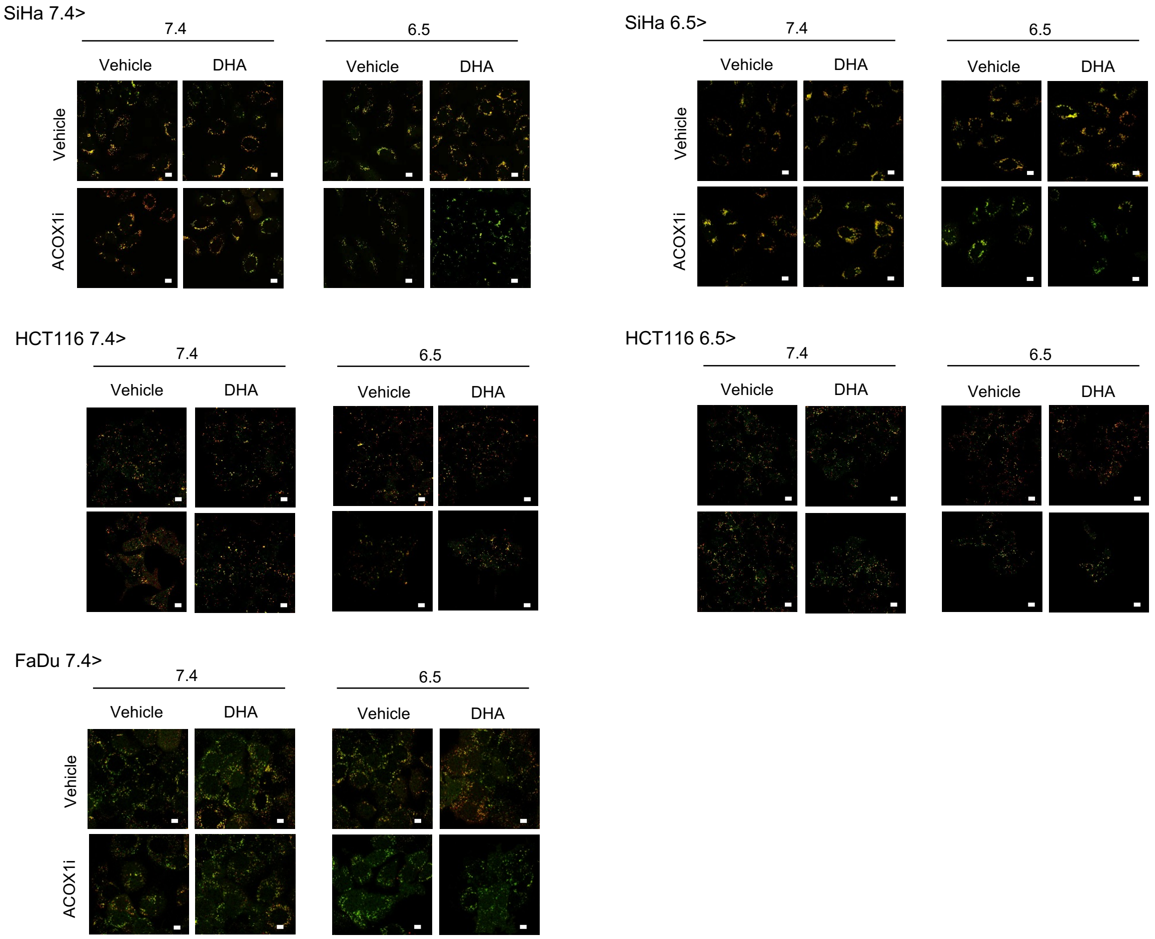
SiHa 7.4>
SiHa 6.5>
HCT116 7.4>
HCT116 6.5>
FaDu 7.4>
7.4
6.5
Vehicle
DHA
Vehicle
ACOX1i

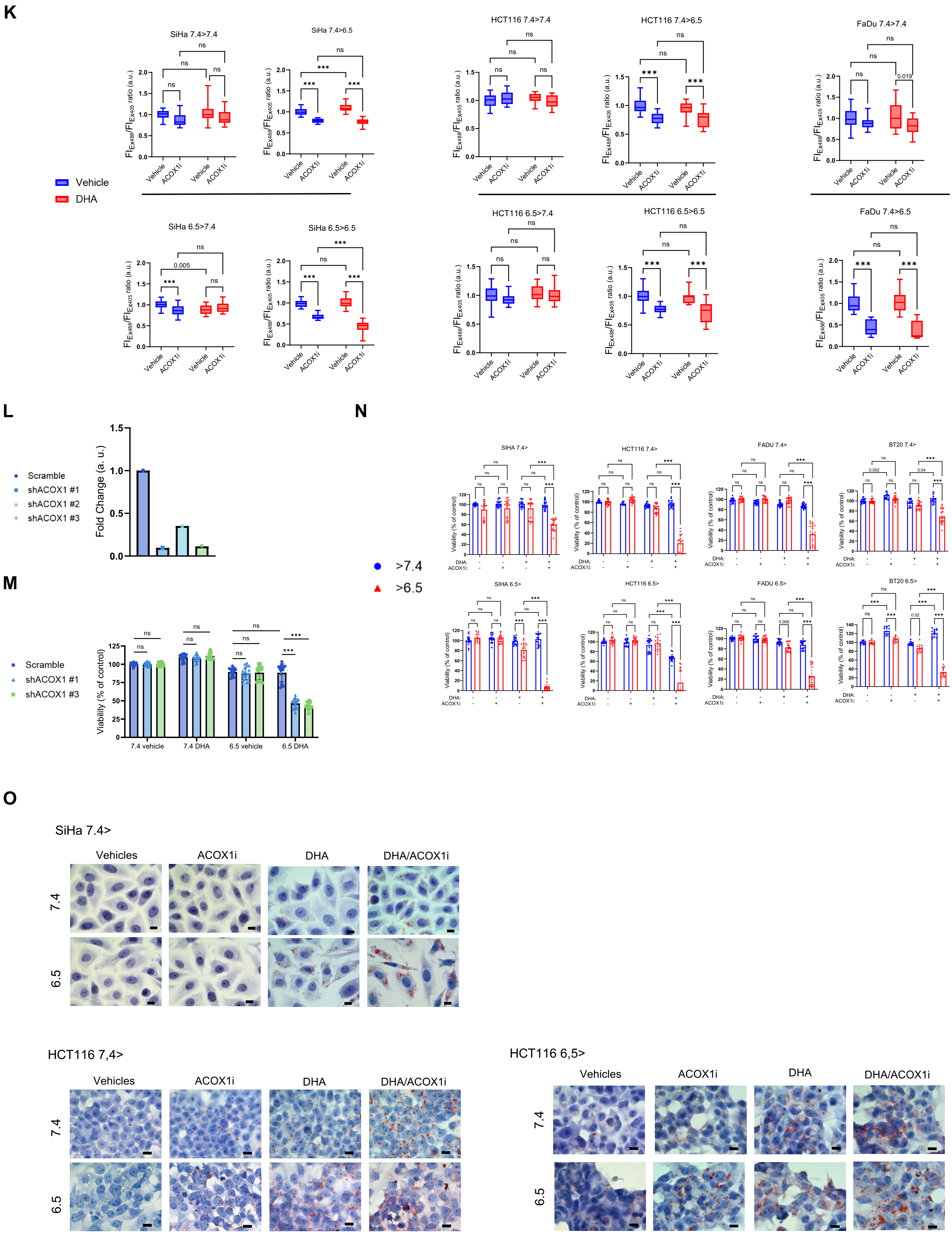
K
SiHa 7.4>7.4
SiHa 7.4>6.5
HCT116 7.4>7.4
HCT116 7.4>6.5
FaDu 7.4>7.4
SiHa 6.5>7.4
SiHa 6.5>6.5
HCT116 6.5>7.4
HCT116 6.5>6.5
FaDu 7.4>6.5
FIEx488/FIEx405 ratio (a.u.)
Vehicle
ACOX1i
Vehicle
DHA
ns
***
0.005
0.019
L
Scramble
shACOX1 #1
shACOX1 #2
shACOX1 #3
Fold Change (a. u.)
M
Scramble
shACOX1 #1
shACOX1 #3
Viability (% of control)
7.4 vehicle
7.4 DHA
6.5 vehicle
6.5 DHA
N
>7.4
>6.5
SIHA 7.4>
HCT116 7.4>
FADU 7.4>
BT20 7.4>
SIHA 6.5>
HCT116 6.5>
FADU 6.5>
BT20 6.5>
Viability (% of control)
DHA:
ACOX1i:
0.002
0.04
0.006
0.02
O
SiHa 7.4>
Vehicles
ACOX1i
DHA
DHA/ACOX1i
7.4
6.5
HCT116 7,4>
HCT116 6,5>

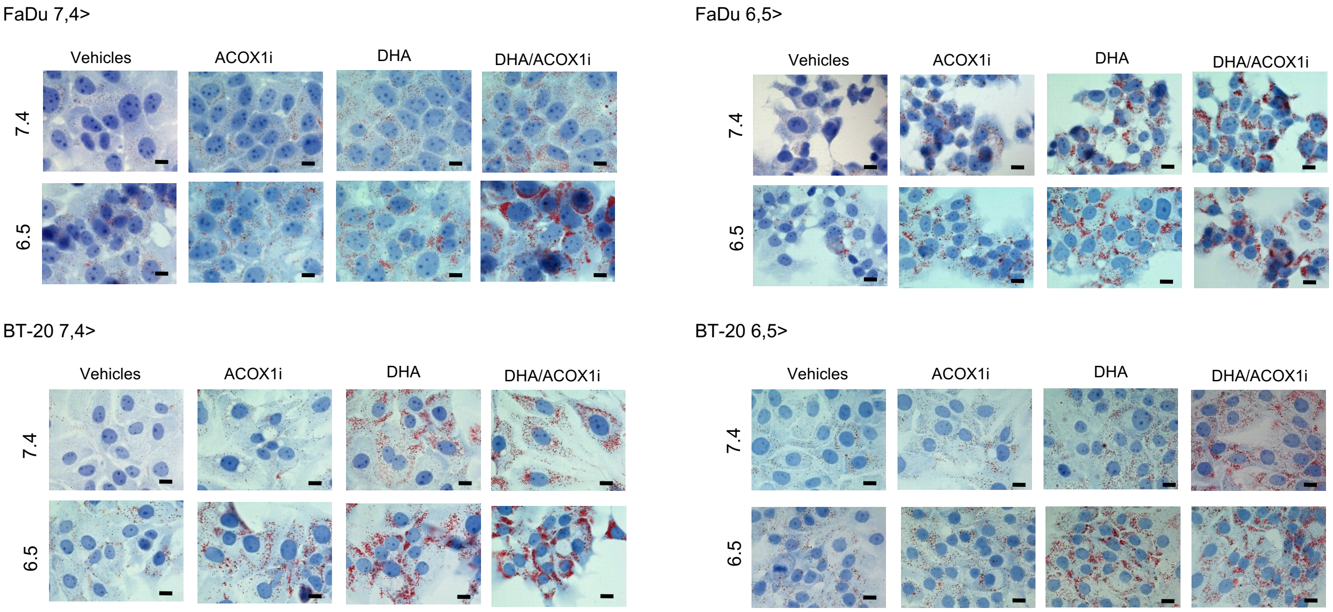
FaDu 7,4>
FaDu 6,5>
Vehicles
ACOX1i
DHA
DHA/ACOX1i
7.4
6.5
BT-20 7,4>
BT-20 6,5>

P

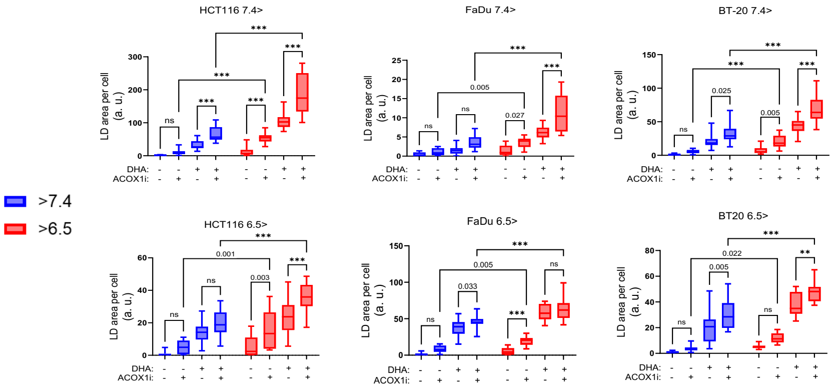
HCT116 7.4>
FaDu 7.4>
BT-20 7.4>
HCT116 6.5>
FaDu 6.5>
BT20 6.5>
LD area per cell (a. u.)
DHA:
ACOX1i:
>7.4
>6.5

Q

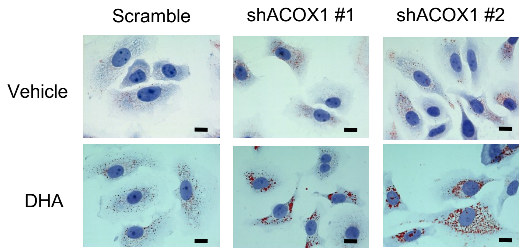
Scramble
shACOX1 #1
shACOX1 #2
Vehicle
DHA

R

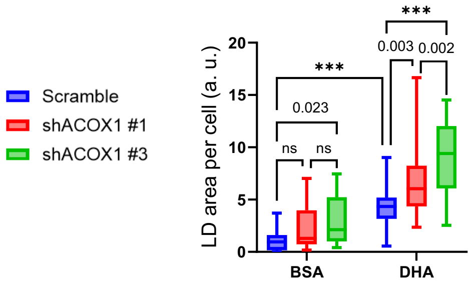
Scramble
shACOX1 #1
shACOX1 #3
LD area per cell (a. u.)
***
0.003
0.002
***
0.023
ns
ns
BSA
DHA

S

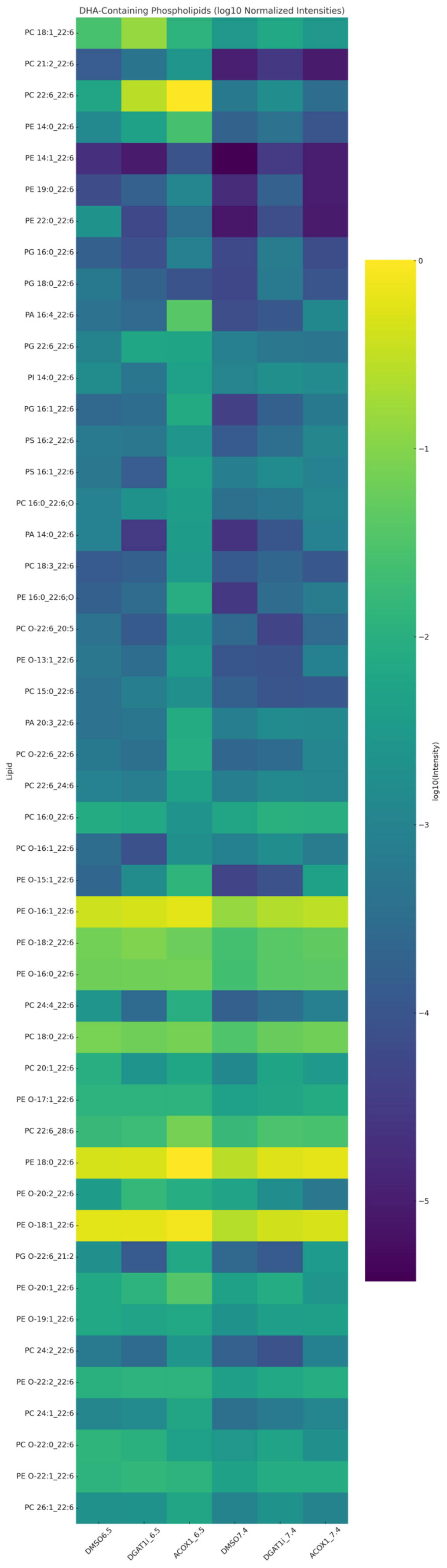
DHA-Containing Phospholipids (log10 Normalized Intensities)
Lipid
PC 18:1_22:6
PC 21:2_22:6
PC 22:6_22:6
PE 14:0_22:6
PE 14:1_22:6
PE 19:0_22:6
PE 22:0_22:6
PG 16:0_22:6
PG 18:0_22:6
PA 16:4_22:6
PG 22:6_22:6
PI 14:0_22:6
PG 16:1_22:6
PS 16:2_22:6
PS 16:1_22:6
PC 16:0_22:6;O
PA 14:0_22:6
PC 18:3_22:6
PE 16:0_22:6;O
PC O-22:6_20:5
PE O-13:1_22:6
PC 15:0_22:6
PA 20:3_22:6
PC O-22:6_22:6
PC 22:6_24:6
PC 16:0_22:6
PC O-16:1_22:6
PE O-15:1_22:6
PE O-16:1_22:6
PE O-18:2_22:6
PE O-16:0_22:6
PC 24:4_22:6
PC 18:0_22:6
PC 20:1_22:6
PE O-17:1_22:6
PC 22:6_28:6
PE 18:0_22:6
PE O-20:2_22:6
PE O-18:1_22:6
PG O-22:6_21:2
PE O-20:1_22:6
PE O-19:1_22:6
PC 24:2_22:6
PE O-22:2_22:6
PC 24:1_22:6
PC O-22:0_22:6
PE O-22:1_22:6
PC 26:1_22:6
DMSO6.5
DGAT1i_6.5
ACOX1_6.5
DMSO7.4
DGAT1i_7.4
ACOX1_7.4
log10(Intensity)
0
−1
−2
−3
−4
−5

Figure S3. Enhanced DHA uptake under acidosis sensitizes cancer cells to peroxisomal inhibition. HCT116, FaDu, and BT-20 cells maintained at the indicated pHe (7.4>7.4 and 6.5>6.5) or following pHe swapping (7.4>6.5 and 6.5>7.4) were supplemented with 50 µM DHA, C22:1, or FA-free BSA as vehicle, and treated as indicated. **A.** Effects of DGAT1 inhibition (15 µM A922500 vs. DMSO, 72h) in the different pHe conditions (N=3, n = 6). **B.** Intracellular pH from pHluorin2 HCT116 cells was measured after a treatment with 15 µM DGAT1i (DMSO as vehicle) with a supplementation of 50 µM DHA (FA-free BSA as vehicle) with the maintenance of pHe at 7.4 (7.4>7.4) or switching at 6.5 (7.4>6.5) during 24h (N3, n=3). **C.** Representative pictures of membrane fluidity in SiHa cells exposed for 48h to 15 µM DGAT1i (DMSO as vehicle) and 50 µM DHA (FA-free BSA as vehicle). **D**. Co-immunofluorescence in fixed acidic-adapted HCT116 of Hyper3-PTS1 targeted to peroxisomes and specific peroxisomal transporter ACBD3. **E-F.** Representative pictures of the merge Hyper3 oxidized and reduced forms in transduced SiHa and HCT116 cells (E) and quantification of Ratio Ex488/Ex405 (reflecting H_2O_2 production) normalised to control in HCT116 cells exposed for 48h to 15 µM DGAT1i (DMSO as vehicle) and 50 µM DHA (FA-free BSA as vehicle) in each pHe condition (N=2, n=10 fields) (F). **G-H.** Same conditions as E and F with 50 µM C22:1 (FA-free BSA as vehicle) (N=3, n=4). **I.** Effects of ACOX1 inhibition (60 µM tricosadiynoic acid vs. DMSO, 24h) in SiHa, HCT116, FaDu, and BT-20 cells with the different pHe conditions and supplementation of 50 µM DHA or C22:1 (FA-free BSA as vehicle) (N=3, n = 6). **J-K**. Representative pictures of the merged Hyper3 oxidized and reduced forms in transduced SiHa, HCT116, and FaDu cells undergoing the indicated pHe changes (J) and quantification of Ratio Ex488/Ex405 (reflecting H_2O_2 production) in HCT116 and FaDu cells exposed for 6h to 60 µM ACOX1i (DMSO as vehicle) in each pHe condition (N=2, n=10 fields) (K). **L.** RT-qPCR of ACOX1 in shACOX1 SiHa with three different shRNA (N=1). **M.** Effects of ACOX1 knock-down in SiHa supplemented with 75 µM DHA (FA-free BSA as vehicle) at pH 7.4 or 6.5 (72 h) (N=3, n = 6). **N.** Effects of mild ACOX1 inhibition (30 µM tricosadiynoic acid vs. DMSO, 72h) in the different pHe conditions with supplementation of 50 µM DHA (FA-free BSA as vehicle) (N=3, n=6). **O-P.** Representative pictures (O) and quantification (P) of ORO-stained LD in HCT116, FaDu, and BT-20 cells undergoing the indicated pHe changes and exposed to 30 µM ACOX1i (DMSO as vehicle) and 50 µM DHA (FA-free BSA as vehicle) for 48 h. Data are normalized to the control condition (N=2, n=10). **Q-R.** Representative pictures (Q) and quantification (R) of ORO-staining LD during 50 µM DHA supplementation (FA-free BSA as vehicle) in the knock-down ACOX1 SiHa cells. Data are normalized to Scramble shRNA supplemented with vehicle (N = 3, n = 10). **S.** Heatmap representing DHA-containing phospholipid species, expressed as mean normalized intensities on a log10 scale (N=3). All scale bars: 10 µm. Graphs are presented as mean ± SD (A, I, M, N) or min to max whisker plots, range = Q1 to Q3, and line = median (B, F, H, K, P, R). N = biological replicates, and n = technical replicates. ns = non-significant; *** = p<0.001. Significance was determined by two-way ANOVA with Turkey's multiple comparison test.

Suppl. Figure 4.

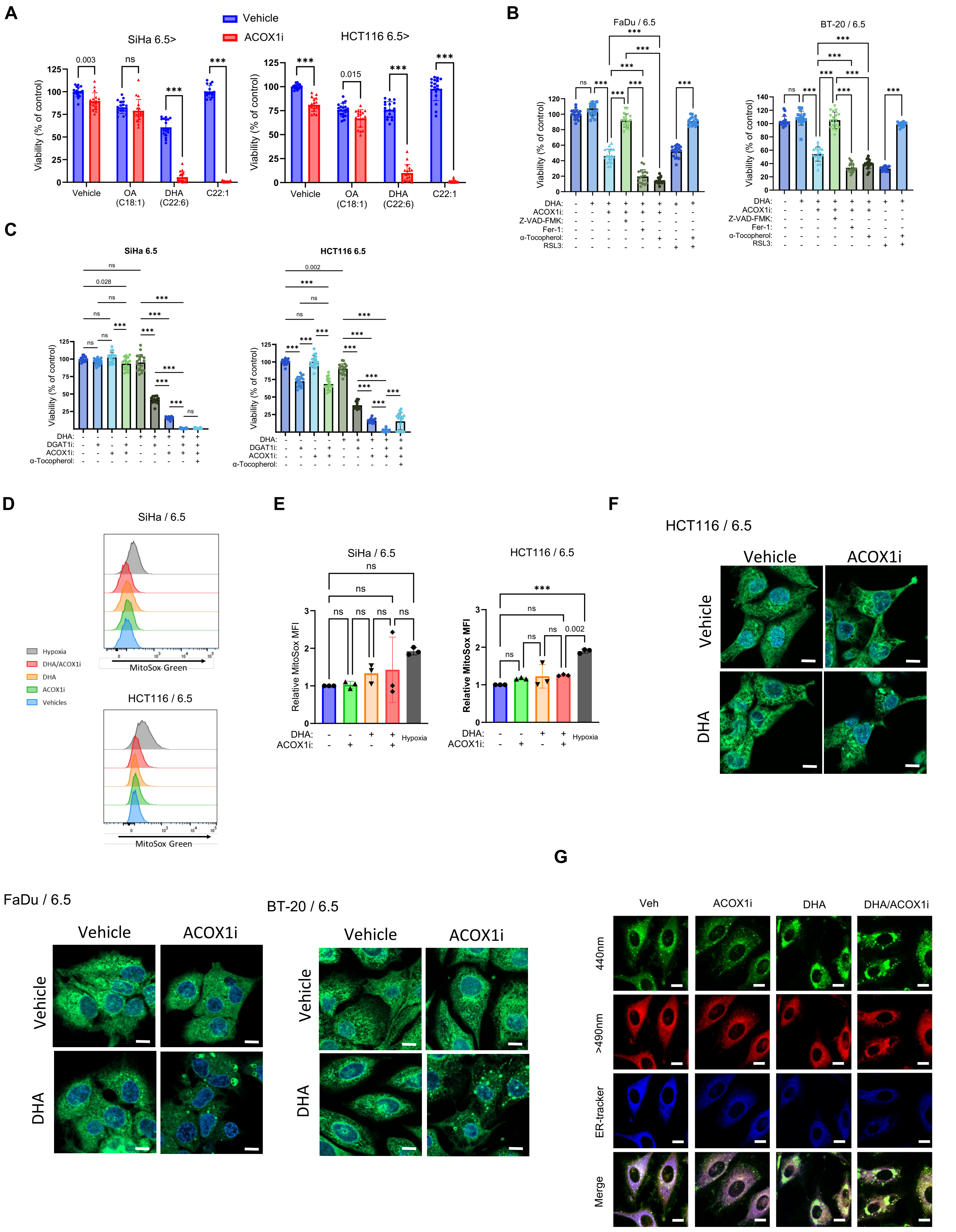

H

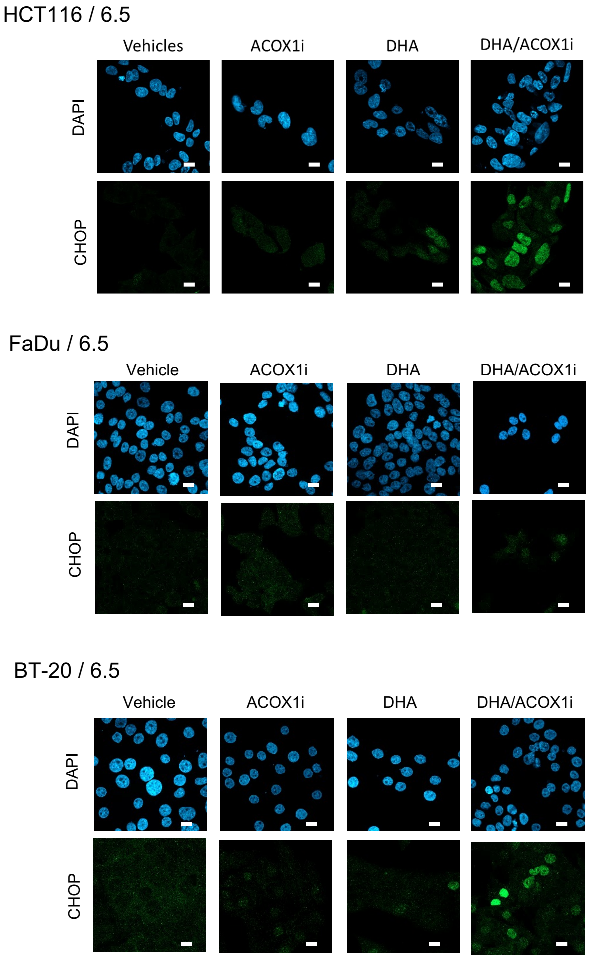
HCT116 / 6.5
Vehicles
ACOX1i
DHA
DHA/ACOX1i
DAPI
CHOP
FaDu / 6.5
Vehicle
ACOX1i
DHA
DHA/ACOX1i
DAPI
CHOP
BT-20 / 6.5
Vehicle
ACOX1i
DHA
DHA/ACOX1i
DAPI
CHOP

I

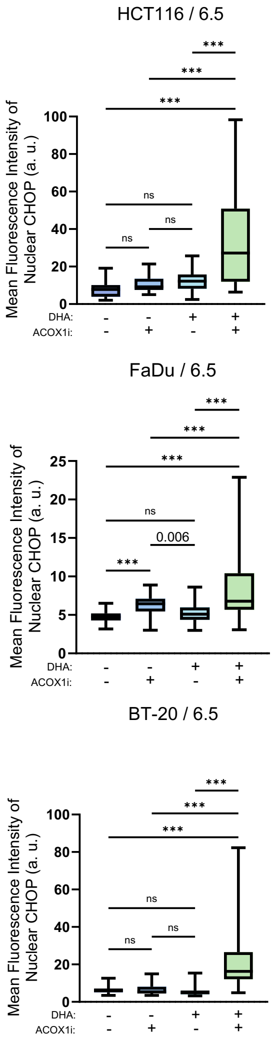
HCT116 / 6.5
Mean Fluorescence Intensity of Nuclear CHOP (a. u.)
***
***
***
ns
ns
ns
0
20
40
60
80
100
DHA: - - + +
ACOX1i: - + - +
FaDu / 6.5
Mean Fluorescence Intensity of Nuclear CHOP (a. u.)
***
***
***
ns
0.006
***
0
5
10
15
20
25
DHA: - - + +
ACOX1i: - + - +
BT-20 / 6.5
Mean Fluorescence Intensity of Nuclear CHOP (a. u.)
***
***
***
ns
ns
ns
0
20
40
60
80
100
DHA: - - + +
ACOX1i: - + - +

J

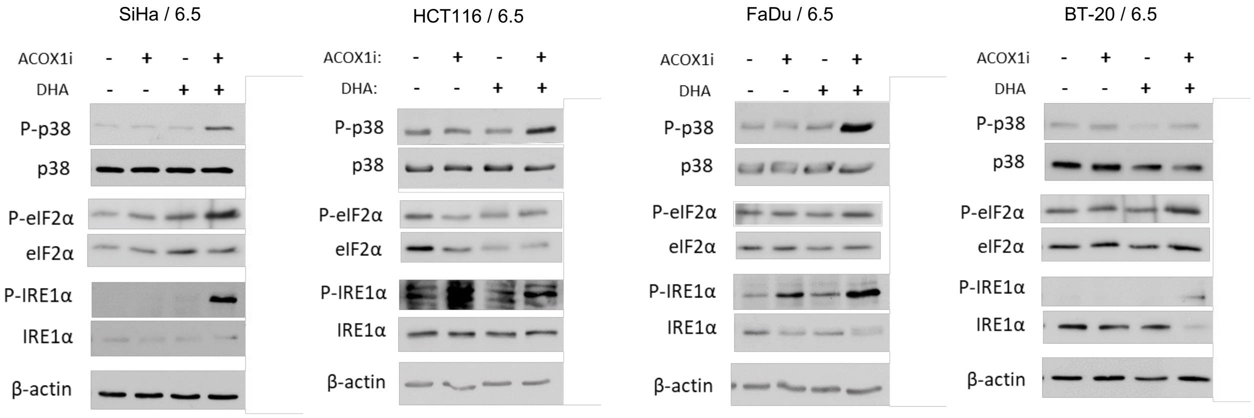
SiHa / 6.5
ACOX1i - + - +
DHA - - + +
P-p38
p38
P-eIF2α
eIF2α
P-IRE1α
IRE1α
β-actin
HCT116 / 6.5
ACOX1i: - + - +
DHA: - - + +
P-p38
p38
P-eIF2α
eIF2α
P-IRE1α
IRE1α
β-actin
FaDu / 6.5
ACOX1i - + - +
DHA - - + +
P-p38
p38
P-eIF2α
eIF2α
P-IRE1α
IRE1α
β-actin
BT-20 / 6.5
ACOX1i - + - +
DHA - - + +
P-p38
p38
P-eIF2α
eIF2α
P-IRE1α
IRE1α
β-actin

K

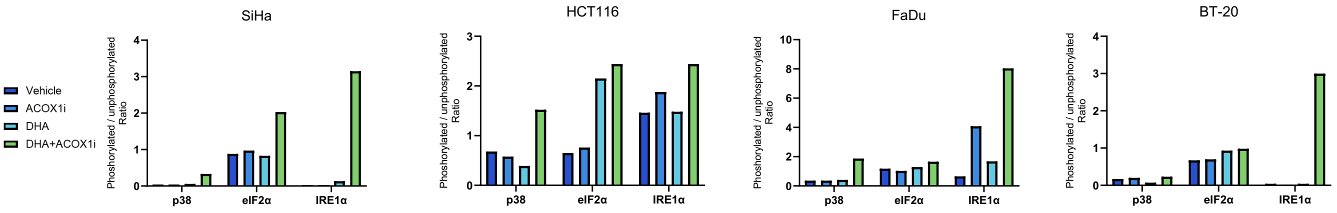
Vehicle
ACOX1i
DHA
DHA+ACOX1i
SiHa
Phoshorylated / unphosphorylated Ratio
0
1
2
3
4
p38
eIF2α
IRE1α
HCT116
Phoshorylated / unphosphorylated Ratio
0
1
2
3
p38
eIF2α
IRE1α
FaDu
Phoshorylated / unphosphorylated Ratio
0
2
4
6
8
10
p38
eIF2α
IRE1α
BT-20
Phoshorylated / unphosphorylated Ratio
0
1
2
3
4
p38
eIF2α
IRE1α

L

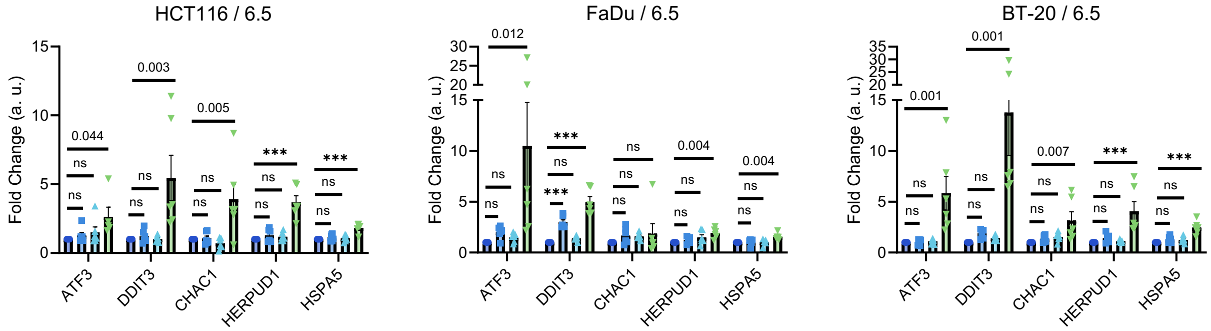
HCT116 / 6.5
FaDu / 6.5
BT-20 / 6.5
Fold Change (a. u.)
ATF3
DDIT3
CHAC1
HERPUD1
HSPA5
0.044
0.003
0.005
0.012
0.004
0.001
0.007
ns
***

M

HCT116 / 6.5

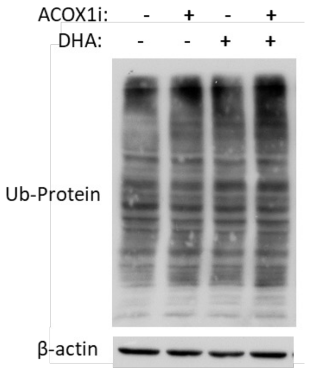
ACOX1i: - + - +
DHA: - - + +
Ub-Protein
β-actin

Figure S4. DHA and ACOX1i combination induce apoptosis without triggering ferroptosis. SiHa, HCT116, FaDu, and BT-20 cells maintained at acidic pHe 6.5 were supplemented with 50 µM DHA or FA-free BSA as vehicle, and treated as indicated. **A.** Viability of acidic-adapted SiHa and HCT116 cells treated with 50 µM OA, DHA, or C22:1 (FA-free BSA as vehicle) and 60 µM ACOX1i (DMSO as vehicle) during 24 h (N=3, n = 6). **B.** Effect of cell death inhibitors (30 µM Z-VAD-FMK for apoptosis or 30 µM Ferrostatin-1 and 20 µM α-tocopherol for ferroptosis) on the viability of FaDu and BT-20 cells exposed to 40 µM ACOX1i, or 5 µM RSL3 as positive control of ferroptosis (DMSO as vehicle) (N=3, n = 6). **C.** Effect of the combination of 15 µM DGAT1i and 30 µM ACOX1i (DMSO as vehicle) on acidic SiHa and HCT116 after 24 h, 20 µM α-tocopherol was used as ferroptosis inhibitor (N=3, n=6) **D-E.** Representative flow cytometry histograms (D) and quantification (E) of mitochondrial ROS in SiHa and HCT116 cells treated for 5 h with 60 µM ACOX1i, followed by 24 h of hypoxia and reoxygenation, were used as a positive control (N=3, n=1). **F.** Live cell confocal microscopy assessment of ER morphology in HCT116, FaDu, and BT-20 upon exposure for 5 h to 60 µM ACOX1i, as determined using 1 µM ER-tracker green reporter. **G.** Representative pictures of membrane fluidity (2 µM Laurdan) in SiHa cells exposed for 24h to 15 µM ACOX1i (or DMSO as vehicle), co-stained with 1 µM ER-tracker red **H-I**. Representative pictures (H) and quantification (I) of nuclear CHOP immunofluorescence in fixed HCT116, FaDu, and BT-20 cells treated for 4 h with 60 µM ACOX1i (n= 70, 110, and 110 independent cells, respectively). **J.** Immunoblotting of total and phosphorylated forms of p38, eiF2α, and IRE1α in HCT116, FaDu, and BT-20 cells treated for 5 h with 60 µM ACOX1i (DMSO as vehicle) and 50 µM DHA (FA-free BSA as vehicle). **K.** The phosphorylated over total intensity ratio for each protein of the previous immunoblotting (N=1). **L-M.** RT-qPCR of genes associated with UPR in HCT116, FaDu, and BT-20 cells (L) and immunoblotting of ubiquitinylated proteins in HCT116 (M) treated for 5 h with 60 µM ACOX1i (DMSO as vehicle) and 50 µM DHA (FA-free BSA) treatments (N=6). All scale bars: 10 µm. Graphs are presented as mean ± SD (A, B, C, E), mean ± SEM (L), or min to max whisker plots, range = Q1 to Q3, and line = median (I). N = biological replicates, and n = technical replicates. ns = non-significant; *** = $p<0.001$. Significance was determined by one-way ANOVA (E, I, L) and two-way ANOVA (A, B, C) with Turkey's multiple comparison test.

Suppl. Figure 5.

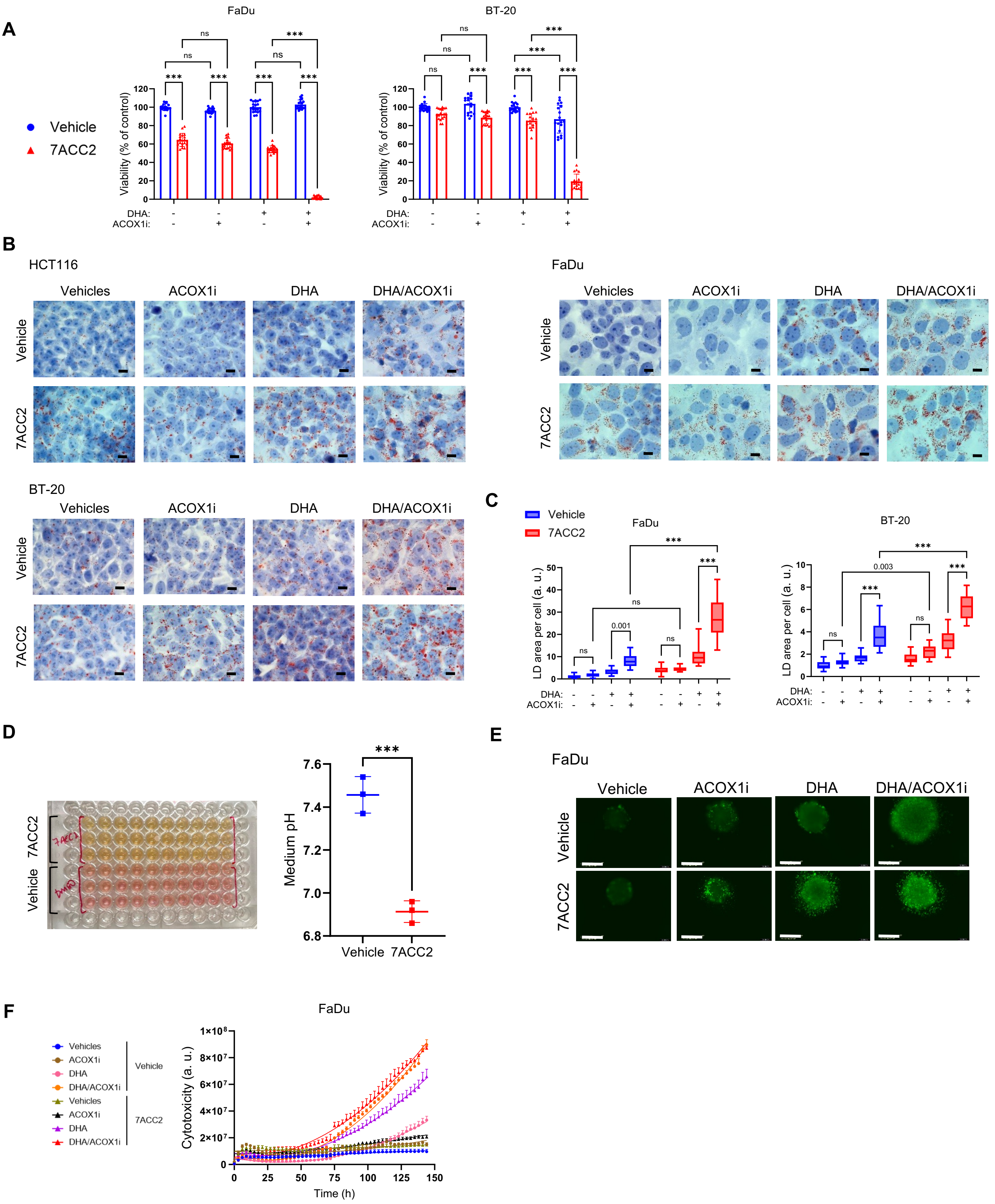

Figure S5. Acidosis-inducing MPCi enhances the cytotoxic effects of DHA supplementation. Cancer cells, spheroids, and organoids were pre-challenged with 10 µM 7ACC2 (DMSO as vehicle) to stimulate glycolysis and induce medium acidification, and treated as indicated with ACOX1 inhibitor (tricosadiynoic acid) and DHA (DMSO and FA-free BSA as vehicle, respectively). **A.** Effects of combining 75 µM DHA and ACOX1 inhibition (60 µM, 72h) on the viability of FaDu and BT-20 cells under induced acidification (N=3, n = 6). **B-C.** Representative pictures (B) and quantification (C) of ORO-stained LD in FaDu and BT-20 cells under induced acidification and exposed to 50 µM DHA and 30 µM ACOX1i. Data are normalized to control (N = 2, n = 10 fields). Scale bar: 10 µm. **D.** pH quantification of organoid media after stimulation of glycolysis by 10 µM 7ACC2. Representative picture of a plate after treatment (left) and quantification (right) (N=3). **E-F.** Representative pictures (E) and quantification (F) of cytotoxic effects measured by Cytotox Green Reagent of combining 100 µM DHA and 60 µM ACOX1i in FaDu spheroids under induced acidification (N=3). Scale bar: 300 µm. Graphs are presented as mean ± SD (A, D), min to max whisker plots, range = Q1 to Q3, and line = median (C) or mean ± SEM (F). N = biological replicates, and n = technical replicates. ns = non-significant; *** = $p<0.001$. Significance was determined by two-way ANOVA with Tukey's multiple comparison test (A, C) and unpaired t-test (D).

Suppl. Figure 6.

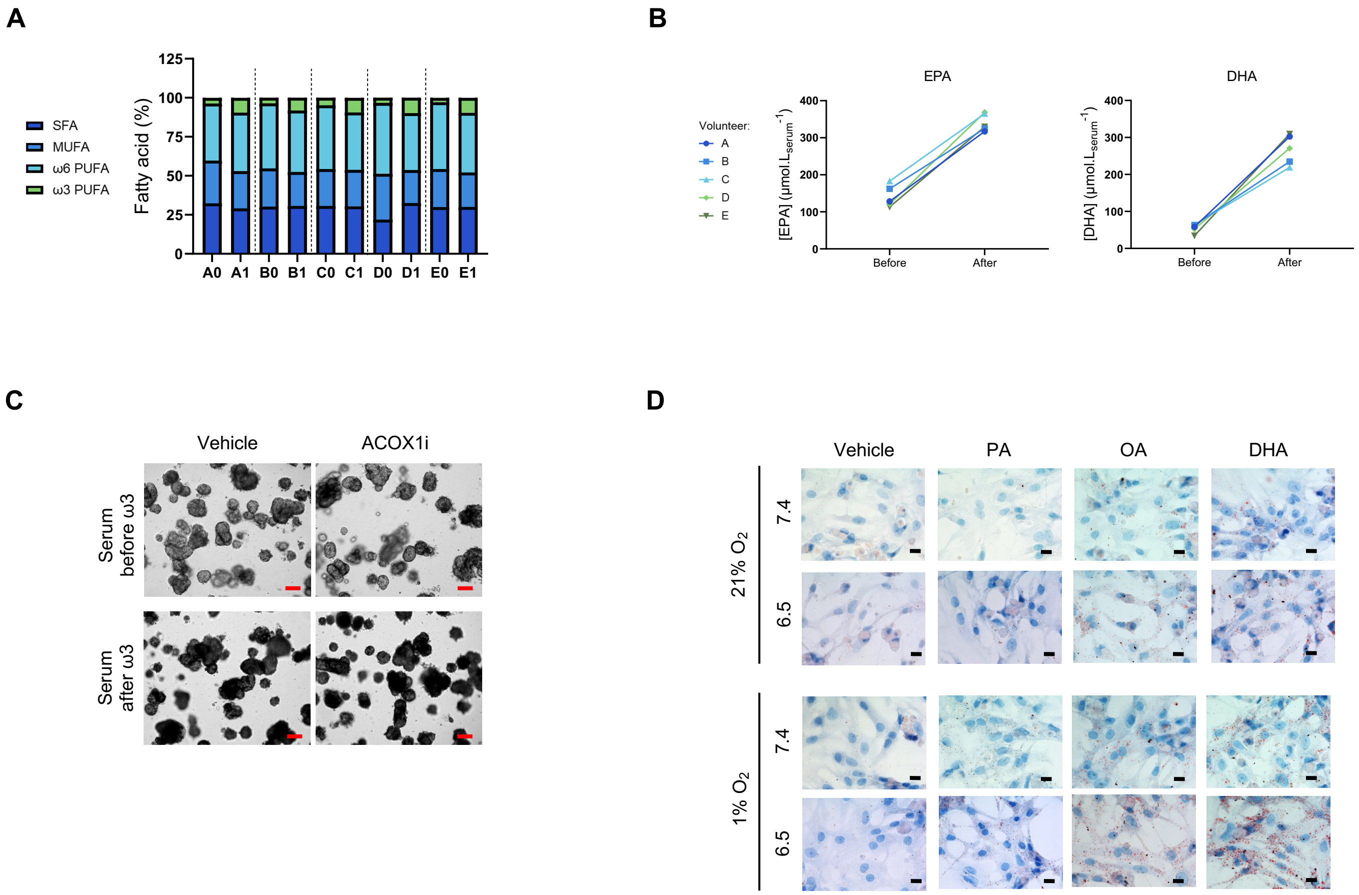

Figure S6. Acidosis enhances the detrimental effects of combining dietary ω3-PUFA and ACOX1 and promotes FA uptake in non-cancer cells. A. Quantification by Gas Chromatography of FA species in human serums from 5 healthy donors, before (0) and after (1) 1-week supplementation with ω3 FAs. **B.** Difference of concentrations of ω3 FAs, EPA and DHA, in the human serum previously described before and after supplementation. **C.** Representative pictures of colorectal cancer organoids treated with ACOX1 inhibitor or vehicle (DMSO) and supplemented with human serum from 5 healthy donors, before and after 1-week supplementation with ω3 FAs. Scale bar: 100 µm. D. Representative pictures of LD in cardiomyocytes cultured at different pH, different pO_2, and supplemented with 50 µM of FA (FA-free BSA as vehicle). Scale bar: 10 µm.

Table S1. **Lentiviral sequences used to transduce cell lines (knockdown or overexpression)**

Purpose	Target gene	Sequence 5' - 3'
Knock down (shRNA)	ACOX1 #1	TTGCAGTGGTCTTCCAAATAT
	ACOX1 #2	ACTACTGAGTAGTTGATTATT
	ACOX1 #3	GCAGCCAGATTAGTAGAAATT
Over-expression	HyPer3-PTS1	ATGGAGATGGCAAGCCAGCAGGGCGAGACGATGTCCGGACCGCTGCACATT<br>GGTTTGATTCCCACAGTTGGACCGTACCTGCTACCGCATATTATCCCTATGCTG<br>TACCAGACCTTTCCAAAGCTGGAAATGTATCTGCATGAAGCACAGACCCACCA<br>GTTACTGGCGCAACTGGACAGCGGCAAACTCGATTGCGTGATCCTCGCACTG<br>GTGAAAGAGAGCGAAGCATTCATTGAAGTGCCGTTGTTTGATGAGCCAATGT<br>TGCTGGCTATCTATGAAGATCACCCGTGGGCGAACCGCGAATGCGTACCGAT<br>GGCCGATCTGGCAGGGGAAAAACTGCTGATGCTGGAAGATGGTCACTGTTTG<br>CGCGATCAGGCAATGTCCGCCGGCTACAACAGCGACAACGTCTATATCATGG<br>CCGACAAGCAGAAGAACGGCATCAAGGCCAACTTCAAGATCCGCCACAACGT<br>CGAGGACGGCAGCGTGCAGCTCGCCGACCACTACCAGCAGAACACCCCCATC<br>GGCGACGGCCCCGTGCTGCTGCCCGACAACCACTACCTGAGCTTCCAGTCCGT<br>CCTGAGCAAAGACCCCAACGAGAAGCGCGATCACATGGTCCTGCTGGAGTTC<br>GTGACCGCCGCCGGGATCACTCTCGGCATGGACGAGCTGTACAACGTGGATG<br>GCGGTAGCGGTGGCACCGGCAGCAAGGGCGAGGAGCTGTTCACCGGGGTG<br>GTGCCCATCCTGGTCGAGCTGGACGGCGACGTAAACGGCCACAAGTTCAGCG<br>TGTCCGGCGAGGGCGAGGGCGATGCCACCTACGGCAAGCTGACCCTGAAGC<br>TGATCTGCACCACCGGCAAGCTGCCCGTGCCCTGGCCCACCCTCGTGACCACC<br>CTCGGCTACGGCCTGAAGTGCTTCGCCCGCTACCCCGACCACATGAAGCAGC<br>ACGACTTCTTCAAGTCCGCCATGCCCGAAGGCTACGTCCAGGAGCGCACCATC<br>TTCTTCAAGGACGACGGCAACTACAAGACCCGCGCCGAGGTGAAGTTCGAGG<br>GCGACACCCTGGTGAACCGCATCGAGCTGAAGGGCATCGGCTTCAAGGAGG<br>ACGGCAACATCCTGGGGCACAAGCTGGAGTACAACGGCACCGGTTTCTGTTT<br>TGAAGCCGGGGCGGATGAAGATACACACTTCCGCGCGACCAGCCTGGAAACT<br>CTGCGCAACATGGTGGCGGCAGGTAGCGGGATCACTTTACTGCCAGCGCTGG<br>CTGTGCCGCCGGAGCGCAAACGCGATGGGGTTGTTTATCTGCCGTGCATTAA<br>GCCGGAACCACGCCGCACTATTGGTCTGGTTTATCGTCCTGGCTCACCGCTGC<br>GCAGCCGCTATGAGCAGCTGGCAGAGGCCATCCGCGCAAGAATGGATGGCC<br>ATTTCGATAAAGTTTTAAAACAGGCGGTTTCCAAGCTCTAA
	pHluorin2	ATGGTGAGCAAGGGCGAGGAGCTGTTCACCGGGGTGGTGCCCATCCTGGTC<br>GAGCTGGACGGCGACGTAAACGGCCACAAGTTCAGCGTGTCCGGCGAGGGC<br>GAGGGCGATGCCACCTACGGCAAGCTGACCCTGAAGTTCATCTGCACCACCG<br>GCAAGCTGCCCGTGCCCTGGCCCACCCTCGTGACCACCCTGAGCTACGGCGT<br>GCAGTGCTTCAGCCGCTACCCCGACCACATGAAGCAGCACGACTTCTTCAAGT<br>CCGCCATGCCCGAAGGCTACGTCCAGGAGCGCACCATCTTCTTCAAGGACGA<br>CGGCAACTACAAGACCCGCGCCGAGGTGAAGTTCGAGGGCGACACCCTGGT<br>GAACCGCATCGAGCTGAAGGGCATCGACTTCAAGGAGGACGGCAACATCCTG<br>GGGCACAAGCTGGAGTACAACTACAACGAGCACCTGGTGTACATCATGGCCG<br>ACAAGCAGAAGAACGGCACCAAGGCCATCTTCCAGGTGCACCACAACATCGA<br>GGACGGCAGCGTGCAGCTCGCCGACCACTACCAGCAGAACACCCCCATCGGC<br>GACGGCCCCGTGCTGCTGCCCGACAACCACTACCTGCACACCCAGTCCGCCCT<br>GAGCAAAGACCCCAACGAGAAGCGCGATCACATGGTCCTGCTGGAGTTCGTG<br>ACCGCCGCCGGGATCACTCACGGCATGGACGAGCTGTACAAGTAA

Table S2. **Primers for qPCR**

Organism	Target gene	Sequence 5' - 3'
Human	RPLP0	For: AGCCCAGAACACTGCTCTC<br>Rev: ACTCAGGATTTCAATGGTGCC
	ACOX1	For: ACAGAGATGGGTCATGGAACTC<br>Rev: TTCCCCTTAGTGATGAGCTGG
	ATF3	For: TCACAAAAGCCGAGGTAGCC<br>Rev: CACTTTCCAGCTTCTCCGACT
	CHAC1	For: GCGCTGTGGATTTTCGGGTA<br>Rev: ACACGGCCAGGCATCTTGT
	DDIT3	For: AGTCTAAGGCACTGAGCGTATC<br>Rev: CTTCCTTCTTGAACACTCTCTCC
	HERPUD1	For: AGAAATCAACGCCAAGGTGG<br>Rev: TCCAGGGGAAGAAAGGTTCC
	HSPA5	For: ACACAGTGGTGCCTACCAAG<br>Rev: TGTCAGGGGTCTTTCACCTTC
